# Cooperativity among Short Amyloid Stretches in Long Amyloidogenic Sequences

**DOI:** 10.1371/journal.pone.0039369

**Published:** 2012-06-22

**Authors:** Lele Hu, Weiren Cui, Zhisong He, Xiaohe Shi, Kaiyan Feng, Buyong Ma, Yu-Dong Cai

**Affiliations:** 1 Institute of Systems Biology, Shanghai University, Shanghai, People’s Republic of China; 2 CAS-MPG Partner Institute of Computational Biology, Shanghai Institutes for Biological Sciences, Chinese Academy of Sciences, Shanghai, People’s Republic of China; 3 Department of Chemistry, College of Sciences, Shanghai University, Shanghai, People’s Republic of China; 4 Institute of Health Sciences, Shanghai Institutes for Biological Sciences, Chinese Academy of Sciences and Shanghai Jiao Tong University School of Medicine, Shanghai, People’s Republic of China; 5 Shanghai Center for Bioinformation Technology, Shanghai, China; 6 Basic Science Program, SAIC – Frederick, Center for Cancer Research Nanobiology Program, National Cancer Institute-Fredeick, National Institute of Health, Frederick, Maryland, United States of America; University of Akron, United States of America

## Abstract

Amyloid fibrillar aggregates of polypeptides are associated with many neurodegenerative diseases. Short peptide segments in protein sequences may trigger aggregation. Identifying these stretches and examining their behavior in longer protein segments is critical for understanding these diseases and obtaining potential therapies. In this study, we combined machine learning and structure-based energy evaluation to examine and predict amyloidogenic segments. Our feature selection method discovered that windows consisting of long amino acid segments of ∼30 residues, instead of the commonly used short hexapeptides, provided the highest accuracy. Weighted contributions of an amino acid at each position in a 27 residue window revealed three cooperative regions of short stretch, resemble the β-strand-turn-β-strand motif in A-βpeptide amyloid and β-solenoid structure of HET-s(218–289) prion (C). Using an in-house energy evaluation algorithm, the interaction energy between two short stretches in long segment is computed and incorporated as an additional feature. The algorithm successfully predicted and classified amyloid segments with an overall accuracy of 75%. Our study revealed that genome-wide amyloid segments are not only dependent on short high propensity stretches, but also on nearby residues.

## Introduction

Amyloid fibrils are polypeptide aggregates that contribute to the complications of many different “protein conformational” diseases [1], [2], [3]. The location of the amyloid deposits varies and typically determines the observed symptoms. In some important neurodegenerative diseases [1], [2], [3], [4], [5] such as Alzheimer’s disease (AD), Parkinson's disease (PD), motor neuron disease and the ‘prion’ dementias [6], these deposits are found in the brain cells and result in dementia. Alternatively, the deposits can occur in the eye lens, leading to the impairment of len transparency, potentially cataract formation and, ultimately, the loss of sight [7]. Thus, it is of fundamental medical interest to understand the mechanisms of fibrillogenesis with the ultimate goal of determining the relative toxicity of soluble polymers, protofibrils and mature fibrils, and designing drugs that interfere with, and ideally inhibit, the formation of the toxic species. The successful prediction and determination of the aggregation propensity of polypeptide sequences would be a test of our understanding of molecular mechanisms of the amyloid formation, offering the hope for effective treatments for amyloid illnesses [8]. Interestingly, functional amyloids have been also found, adding the challenges to understand why nature can utilize normal amyloid forming mechanism, and avoiding detrimental amyloid formation.

In the normal soluble conditions and depending upon the microenvironment [9], the amyloidogenic polypeptides may assume different conformations including random coil, α-helices, and β-strands. However, eventually, all amyloid fibrils become dominant β-sheet structure. Often, the aggregation of a protein domain could be trigged by a short protein stretch within the domain, typically a hexapeptide fragment [10], [11]. Consistent with amyloid stretch hypothesis, many computational algorithms can be used to screen the short (hexapeptide) fragments to predict amyloidogenicity of protein sequence, with different success rates [12], [13], [14]. Using the crystal structure of NNQQNY as a model system, genome-wide analysis revealed that about 15% of *E. Coli* and 18% human genomes are such segments with high fibrillation propensity, which can be classified as the amylome: the universe of proteins that are capable of forming amyloid-like fibrils [15].

Apparently, not all of the short amyloid stretches are capable to induce host protein aggregation, probably due to nature’s evolution [15], [16]. Experiments have shown that insertion of short amyloid stretches into globular proteins [11], [17], [18] may induce the fused protein to form amyloid. But the conversion of native proteins into amyloid fibrils depends on the sequence context of the inserted short amyloid stretches. Thus it is important to understand the pattern of short amyloid stretches within longer amyloidogenic segments, which presents major challenges to both the experimentalist and the theoretician. Much of the work encounters an empirical obstacle due to the experimental complexities; the sensitivity of protein aggregation to the slightest change in protein amino acid composition, solvent properties, or protein concentration; and the lack of robust theoretical models of misfolding and aggregation.

In this work, in order to understand the context dependent protein aggregation, we developed a method that correlates the amyloidogenicity of an amino acid at a given position with all other amino acids in a long sequential segment. Three steps are taken to achieve optimal characterization of known amyloidogenic sequences. In the first step, we have used multivariate statistical analyses of a large number of amino acid features to correlate with the amyloid formation. Based on the results from the preliminary feature analysis, we developed an algorithm to search for the low energy structures in a long amino acid segment. Finally, the energy terms was incorporated into feature selection algorithm to refine amyloid sequences characterization and genomic wide sequences search for possible amyloid sequences. We found that, within a 27 residues long segment, the amyloidogenicity of short amyloid stretch also has cooperative contributions from two distant regions in N-terminal and C-terminal directions. Our work has provided interesting insights into the complex process of fibril aggregation, extend the evaluation of physicochemical properties contribution to the differential aggregation behavior of fibril polypeptides.

## Result and Discussion

### Initial Feature Analysis of Physical and Chemical Properties of Amino Acids in Amyloid Formation

Each peptide chain is represented by 918 features. The first step to select features important to amyloid formation is the feature pre-evaluation using mRMR program, which was downloaded from website http://research.janelia.org/peng/proj/mRMR/index.htm. The result of mRMR is a table called mRMR list records the feature indices. Besides the mRMR list, the mRMR program will also output a list called MaxRel list, which contains the relevance of all features with the class variable. Both mRMR and MaxRel list all the features in the output for the following-up selection procedures. For the results of mRMR and MaxRel in this paper, please see Table S1 and S2 for more information.

**Figure 1:**
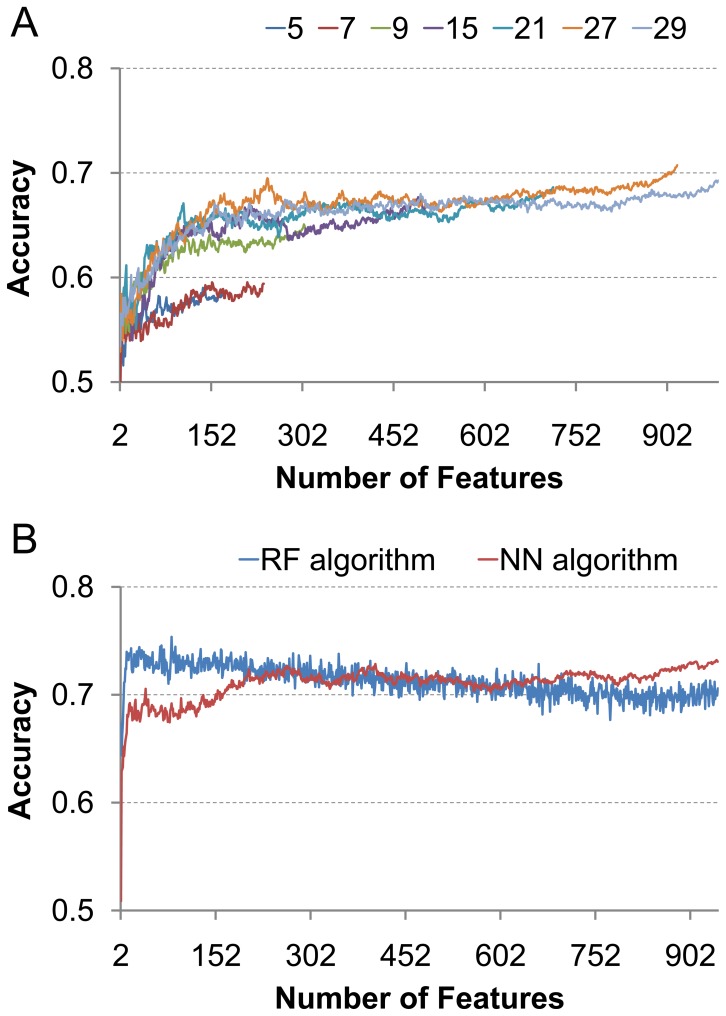
Context dependent behavior of amyloid formation can be shown from the change of prediction rate with window size. (A) Accuracy of prediction increases with the length of window size and maximized at a 27 residue segment. (B) Random forest (RF) algorithm removed the redundancy among the features and increase prediction accuracy.

In order to obtain the optimal feature set, 918 candidates nearest neighbor (NN) models were built for the incremental feature selection (IFS) procedure and Table S3 is the accuracy of each model. The highest overall accurate rate of IFS is showed in Figure 1A. The highest overall accurate rate of IFS reached 70.7% with all the 918 features selected in the feature set. As the optimal dataset contained all the features we used, the selection of the features with contribution to the accuracy were carried out.

**Figure 2:**
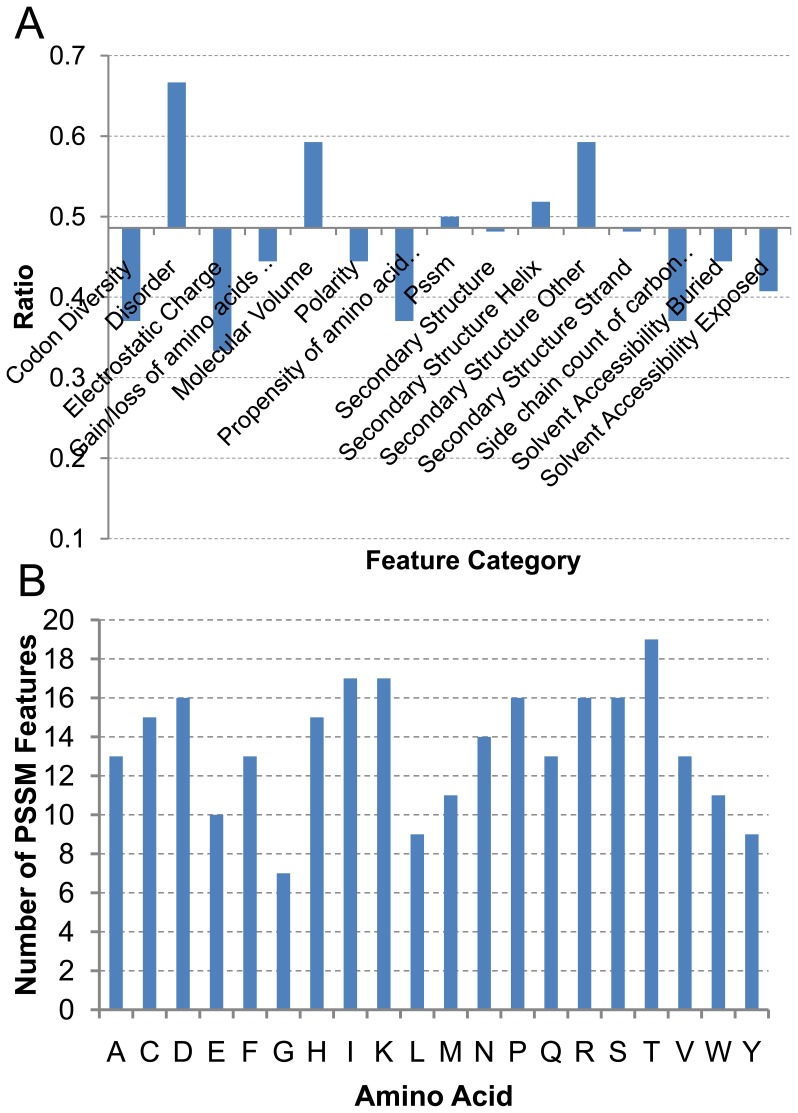
Feature analysis revealed important factor for amyloid formation. (A) The ratio of each feature category occurred in the selected 446 features in the optimal set compared to the ratio of 48.6% which is the ratio of selected features out of the total number. the disordered factors contribute most to the fibril formation followed by the secondary structure factors, amino acid volume factors and pssm factors. (B) the pssm features of each amino acid contained in the selected 446 features.

As the IFS result showed in Figure 1A, the accuracy fluctuates when 200–600 features are used, indicating that the addition of some features makes the accuracy decreased. Although the optimal feature set contains all the 918 features, we select these features that increase the accuracy for further analysis, since they are more relevant to amyloid formation. The further analysis of the feature enrichment results in 446 features, which are 48.6% of the feature number in the optimal set. The details of all the 446 features are listed in the Table S4. In Figure 2A we highlight the ratio of each feature category occurred in the selected 446 features in the optimal set. We use the ratio of 48.6% as a reference ratio since it is the ratio of selected features out of the total number. It can be seen from Figure 2A that the disordered factors contribute most to the fibril formation followed by the secondary structure factors, amino acid volume factors and pssm factors.

All three factors of disorder, secondary structure, and amino acid volume are related to protein folding and packing density upon amyloid fibril formation. The amyloid fibril formation comes as either unfolding of globular protein or perturbation of natively disordered proteins. The subtle changes of the balance of forces in folded protein may lead to misfolded states and aggregated proteins [19], [20]. Thus it is easily understandable that amino acid disorder feature contribute mostly. Amyloid fibrils are dominated with β-sheet conformation. The β-pleated sheet, the building block of amyloid fibers, was suggested to be the thermodynamically most stable arrangement of all the possible peptide dimers and oligomers both in vacuum and in aqueous environments [21]. The β-sheet conformation can be formed by secondary structure change of α-helices or directly from β-sheet domains with disulphide bonds constraints [22], [23]. The contribution of amino acid volume could be that the tight packing of side-chain chains to form zipper structure between β-sheet is very important to the stabilization of amyloid fibril structure [24], [25], [26].

As shown in Figure 2B, the contributions of pssm features reflect the overall propensity of each amino acid in amyloid fibril formation. The conventional wisdom is that hydrophobic/aromatic residues are important to stabilize amyloid fibril [27], [28]. However, our results indicated that the aromatic residues (Trp, Phe, Tyr) are not necessarily having the high tendency to form amyloid. Among the three amino acids with highest propensity (Ile, Thr, and Lys), only the Ile is hydrophobic. Isolecine has the highest propensity to form amyloid in A-β peptide related sequences [29]. It was proposed that nature tends to avoid Ile conservation in protein-protein interactions to avoid amyloid formation [16]. Within the 20 amino acid, threonine (T) seems to be the highest proximal amino acid in fibril formation. This is consistent with the secondary structure factors contribute much to the fibril forming, as threonine is strongly related to the β-secondary structure. However, it is interesting to see that positively charged Lys is among the top three amino acids with highest amyloidogenic propensity. The reason could be that (1) the peptide segments with Lys have higher disorder tendency, and (2) Lys is important for certain structural features in amyloid formation.

### The Cooperativity Among the Short Amyloid Stretches is Consistent with the Common Motif in Amyloidogenic Structure

Most previous works used segments with small length in amyloid prediction. Here we examine the effects of different lengths used in amyloid prediction. Our algorithm explores the context dependent features in amyloid formation, and amyloid formation propensity of residue at position i is also dependent on the sequences of i-j and i+j, when j is the length of segments in N-side and C-side of position i. Thus the overall length of segment is 2j+1 in our study. We systematically searched the optimal length of sequential segment used in our amyloid prediction from 5 to 31. In Figure 1A, we selectively report the results of 7 of them. Essentially, we found that the overall prediction accuracy by Nearest Neighbor model increases with the increasing length of sequential segment, and peaked at length of 27 residues. Our results demonstrated that the most likely amyloidogenic sequence segment in a protein is around 30 residues.

**Figure 3:**
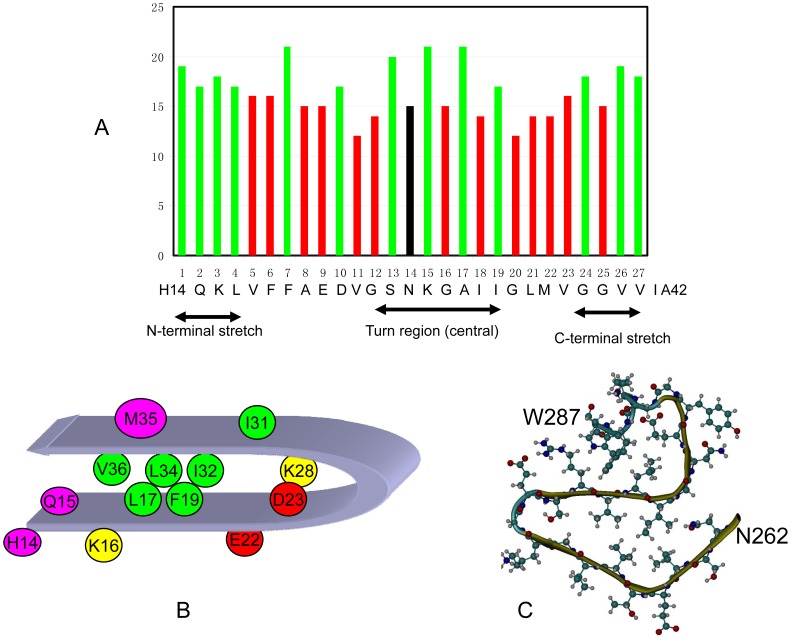
The cooperativity among the short amyloid stretches is consistent with the common motif in amyloidogenic structure. (A) Weighted contributions of an amino acid at each position in the 27 residue segment revealed three regions. The contribution of each position is measured by the number of features in each position. The average contribution from all positions is 16.5. The positions with contributions higher than average are in green, and the red bars are position with contribution less than average. The 14^th^ residue in the center is highlighted as black. The arrangement of the three regions are similar to the common motifs of amyloid structures of β-strand-turn-β-strand motif in A-β peptide amyloid (B) and β-solenoid structure of HET-s(218–289) prion (C).

We then examine the relative contribution of each position within the 27 residue segment. Figure 3A plots the contribution at each position to the central amino acid’s amyloidogenicity. The contribution of each position is measured by the number of features in each position. The average contribution from all positions is 16.5. As indicated in Figure 3A, the positions with contributions higher than average are in green, and the red bars are position with contribution less than average. Based on these contributions, we may divide the 27 residues into three regions: the central stretch and two distant stretches in N-terminal and C-terminal directions. Each stretch can be comparable to commonly used short amyloid stretch of hexa-peptide. We can see that tripeptide (positions 13-14-15) contain the central amino acid is among the highest region, indicating that closest local effect. The alternative pattern for positions 15, 17, and 19 may reflect the regular side chain interaction in a typical β-strand. It is important to see the higher contributions from two distant stretches in N-terminal and C-terminal directions, which clearly show that amyloidogenicity of central stretch also depends on sequence context, i.e., cooperatively from N-terminal and C-terminal stretches.

The cooperativity among the three short amyloid stretches may come from the common motifs of amyloid structure. Two typical structures are β-strand-turn-β-strand motif in A-β peptide amyloid and β-solenoid structure of HET-s(218–289) prion [27]. The β-strand-turn-β-strand motif constitutes many fibrillar cores, for example, A-β peptide, amylin, K3 peptide from β2-microglobulin, and prion protein. Previous work [27] has revealed that A-β peptide amyloid is considered to be a representative motif for the β-strand-turn-β-strand motif in Figure 3B. We noticed good correspondence between Figure 3A and A-β peptide structural motifs. As can be seen in Figure 3, the contribution of each position in the 27 residue sized segment indicates that the 7^th^, 13^th^, 15^th^ and 17^th^ positions are the most important in the fibril forming as illustrated in Figure 3B. If we consider that the four positions with highest contributions corresponding to turn region, salt bridging interaction, and hydrophobic core interactions which are all important to stabilize A-β peptide as the bottom part of Figure 3B, the four positions can perfectly match the U-turn structure. The structural features in the 27 residue sized segment is also compatible with other amyloid structural motif, like HET-s(218–289) amyloid fibrils [30], [31]. In Figure 3C, we show the structural motif of the 26 residue segment from HET-s(218–289) amyloid fibrils. In is clear that the structural repeat can be divided into several short stretches as well.

### Coarse-grained Energy Evaluation Based on the βstrand-turn-βstrand Motifs

**Figure 4:**
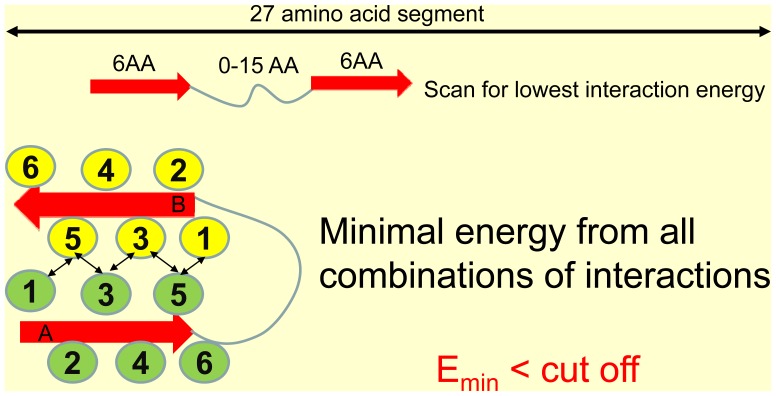
Amyloid interaction energy can be searched by the summation of residue interactions between two short amyloid stretches. The βstrand-turn-βstrand motif is defined as two six-residue β-strands connected with a flexible turn with a length up to 15 residues, with total window length of 27 residues. When there is no linker (L = 0) or the linker is very short (for example, L = 1−2), the motif may be classified as triangular shape observed for β-solenoid structure in Figure 3.

The similarity of the observed features to βstrand-turn-βstrand motif promoted us to develop a structure based algorithm to examine the residue interaction energies in the amyloidogenic sequences. First, we define a possible βstrand-turn-βstrand motif as two six-residue β-strands connected with a flexible turn with a length up to 15 residues (Figure 4). When there is no linker (L = 0) or the linker is very short (for example, L = 1−2), the motif may be classified as triangular shape observed for β-solenoid structure (Figure 3C). Based on the structural motif, we calculate the residue interaction energy:
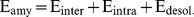
Where E_inter_ is the effective inter-residue contact energy between two adjacent peptides chains; E_intra_ is the effective inter-residue between βstrand A and βstrand B within the same chain; and E_desol_ is the desolvation energy for the residue buried between two β-strands A and B. The delsolvation penalty energies for buried residues were optimized to enlarge the gap between the amyloidogenic sequences and non-amyloidogenic sequences, as in Table 1.

**Table 1:** Delsovation energy penalty.

Amino Acid	Amyloid desolvation penalty
Gly	0
Ala	30
Val	−16
Ile	9
Leu	33
Ser	8
Thr	−3.0
Asp	50
Asn	44
Glu	44
Gln	36
Lys	50
Arg	50
Cys	50
Met	34
Phe	−5.0
Tyr	6.0
Trp	20
His	20
Pro	0

The E_inter_ and E_intra_ are calculated by summing of effective self-contact-potentials developed by Bahar and Jernigan [32].

E_inter_  =  
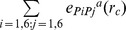
, where the 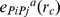 is the effective self-contact-potentials between residue P_i_ and P_j_ in two β-strands with either parallel or anti-parallel registration.

Similarly, E_intra_  =  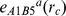
+ 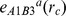
+ 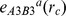
+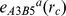
+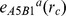 + 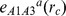
+ 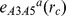
+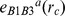
+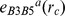, to add the potentials from all intra-chain contact residues (Figure 4A). The possible associations between the two β-strands were exhaustively searched to find the most negative value, which was assigned to the 27 residue segment.

Finally, the residue with the energy lower than the cutoff value (−54.0) were defined as amyloidogenic residue. The number of amino acids in the negative dataset (17102 amino acids) is much more than the number of amino acids in the positive dataset (1370 amino acids). Thus, the accuracy of prediction of negative dataset dominates the accuracy of overall prediction. Therefore, the energy based prediction focus on excluding false positive and maintaining reasonable rate of positive prediction and overall accuracy. After optimizing the value of E_desol_ for all amino acids (Table 1), the accuracies of energy based prediction were 49.5, 84.1 and 81.7 for positive dataset, negative dataset and overall accuracy, respectively.

### Prediction of Amyloid Formation

**Figure 5:**
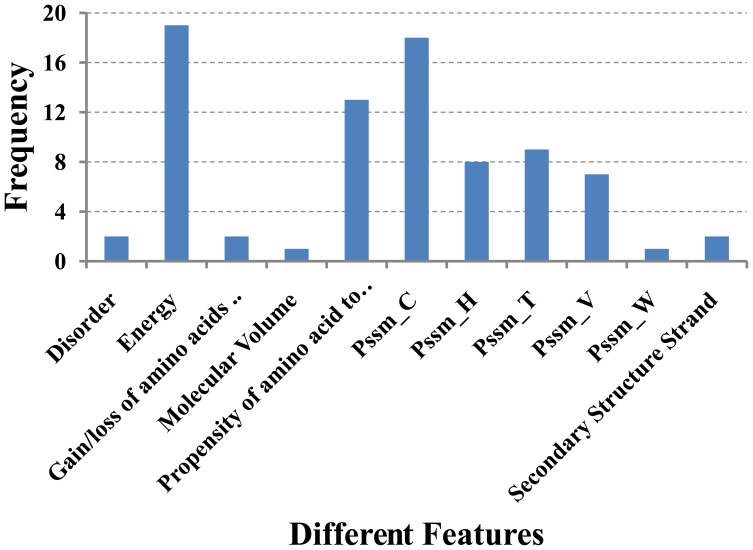
The distribution of different features in the optimal feature set with 82 features indicated the protein-protein interaction energy dominate the amyloid formation. Pssm_C describes the likelihood that the amino acid in the sequence mutates to the cystine (C), Pssm_H describes the likelihood that the amino acid in the sequence mutates to the Histidine (H), and so forth.

With the energies calculated using the β-strand-turn-β-strand motif as additional amino acid features, we repeat the IFS analysis with NN algorithm on our amyloid fibril dataset. Surprisingly, we found that prediction accuracy now is dominated by energies and reaches to 73% with the first 943 features, which can be seen from the blue curve in Figure 1B. Additional algorithm was used to remove the redundancy among the features and to improve the prediction accuracy. Recently, random forest (RF) algorithm [33] has been successfully constructed classifier to tackle various biological classification problems [34], [35], [36]. Therefore, RF was used to replace the NN in the IFS procedure. As shown in the Figure 1B, the highest rate reaches 75% at the first 82 features, much less than the initial 918 features when energy factors are not included, also much less than the 943 features when energy factors are included and NN algorithm is used. The distribution of different features in the optimal feature set with 82 features is shown in Figure 5, from which we know prediction accuracy now is dominated by energies and ten other factors. We list the top 10 contributing features in Table 2. The dominance of energy feature and high success rate indicated that theβ-strand-turn-β-strand motif based algorithm encompassed the essence of amyloid fibril formation.

**Table 2:** The predicted results of IFS procedure with random forest (RF) algorithm based on the first 11 features in optimal features.

Order	Added feature		Accuracy of positivedataset (%)	Accuracy of negativedataset (%)	Overall accuracy (%)
	Amino Acid	Attribute			
1	AA14	Energy	58.91	76.28	67.59
2	AA13	Pssm_C	55.77	78.91	67.34
3	AA27	Disorder	68.10	60.66	64.38
4	AA14	Propensity of amino acid to be conserved at protein-protein interface	71.17	61.39	66.28
5	AA26	Energy	70.66	64.01	67.34
6	AA1	Energy	73.65	64.89	69.27
7	AA26	Pssm_C	75.47	66.79	71.13
8	AA3	Pssm_H	75.91	66.50	71.20
9	AA23	Pssm_H	77.15	67.74	72.45
10	AA18	Energy	78.25	66.50	72.37
11	AA21	Pssm_C	79.42	68.54	73.98

In the table, the “AA14” represents the 14^th^ amino acid residue of the peptide. Pssm_C describes the likelihood that the amino acid in the sequence mutates to the cystine (C), Pssm_H describes the likelihood that the amino acid in the sequence mutates to the Histidine (H).

Using the finalized energy evaluation algorithm and selected 82 other features, we scan yeast *S. cerevisiae* and *E.coli* proteome to examine the percentage of protein segments which are able to form amyloid fibril. The prediction for the yeast *S. cerevisiae* and *E.coli* genome is as below: *E.coli*: 16.39% and yeast: 17.27%; which are close to but lower than the predictions made using only short peptide fragments. Goldschmidt et al. has used a triplet method and 3D based method to search the high propensity (HP) segment for fibrillation. They found that the *E. Coli* may have 15.1% (3D method) to 22% (triplet method) HP segments, while *S. cerevisiae* has about 21.7%. The agreement of the predictions may come from the cancelation of two factors. Due to the context dependent behavior of short amyloid stretch, some of the predicted short HP segments in Goldschmidt’s study may not be able to form amyloid. However, other short amyloid stretches that are not able to be identified independently could be amyloidogenic due to the cooperativity from near residues. Overall, the agreement of our genome-wide prediction and Goldschmidt’s work highlight the significance of ability of protein sequences to form amyloid.

### Conclusion

Soluble proteins may from highly ordered fibril aggregates. Such transitions occur under pathological conditions ranging from neurodegenerative to many other systemic different “protein conformational” diseases. We have studied the long sequential amyloid segments within protein domain by comparing known amyloidogenic sequences with computational predictions.

There are already two types of computational algorithms investigating the aggregation propensity of peptides or proteins and to identify the segments most prone to form fibrils. The first algorithm uses phenomenological models based on the physicochemical properties only for the amino acids to predict each amino acid changes in aggregation rate [37], [38], [39], [40]; the second one combines support vector machine simulations of a protein segment with the micro-structure of short fibril-forming peptides to gain insight into aggregation propensity [12], [13]. Our algorithm combined Position-Specific Scoring Matrices (PSSM) [41], [42], [43] and multivariate statistical analyses of a large number of amino acid attributes to examine the cooperativities among short amyloid stretches within long amyloidogenic sequence segment.

The most important finding from our analysis is that a long segment with about 30 residues, rather than a short amyloid stretch, defines the amyloid forming ability of large protein. Within the long segment, the short amyloid stretch may have synergetic interaction with other short stretches either in N-terminal or C-terminal directions. The cooperativity among the short amyloid stretches may come from the common motifs of amyloid structure such as the U-shape Aβ amyloid and triangular prion amyloid fibrils. Subsequently, an energy evaluation algorithm has been developed based on interactions between the short amyloid stretches in the longer segements. Our approach successfully classified and predicted amyloid formation with overall accuracy of 75%. The prediction of the amylome in the yeast *S. cerevisiae* and *E.coli* genome is consistent with previous study by Goldschmidt et al, but with different molecular mechanism.

Our work extended the concept of amyloid stretch by revealing the context dependent behavior of short amyloid stretch in longer protein sequences. The ability of short amyloid stretch to induce longer protein into eventual amyloid formation depends on the ability of the short amyloid stretch to form compact structure with nearby segment. It is likely that two short amyloid stretches within the long segments would share the consensus structural pattern for amyloid formation for long protein chain [44], represented by the amyloid Aβ peptide sequence pattern found in many other amyloid forming peptides [44].

It has been known that both long rang contacts and local orders are important for islet amyloid polypeptide (amylin) [45], [46]. Many well-known amyloid proteins have several fragments or repeats that are able to aggregate independently or cooperatively. It was still not well understand how these short amyloid stretches cooperatively interact with each other. For example, segment 16–22 and 25–35 of Aβ peptide can effectively hold a β-strand-turn-β-strand motif. Yet, in full length Aβ40 (or Aβ42), mutations at position 1, 10, 20, 30, or 40 (for Aβ40) or 42 (for Aβ42) can all affect amyloid formation [47]. Our currently study provided statistical feature of known wild type amyloidogenic sequences. In the future study, we are going extend the dataset to include experimental information of point mutations, and to predict mutation effects on amyloid formation. Hopefully, our finding of the context dependent behavior of the short amyloid stretches within long amyloidogenic sequences may help to understand many experimental observations.

## Materials and Methods

Based on the previous published collections of amyloidogenic proteins [12], [39], [48], we searched the SwissProt database and obtained 46 protein sequences with 17102 amino acids, in which there are 1370 experimentally verified fibril-forming sites.

In the first step, each peptide chain is represented by 918 features; and 5 physicochemical and biological features of them are taken from AAIndex (http://www.genome.ad.jp/aaindex/), a database of numerical indices representing various physicochemical and biochemical properties. Amino acid disorder score in a protein sequence was calculated using VSL2 [49]. The secondary structure and solvent accessibility scores were obtained using predictors SSpro 4 [50]. We included features of amino acid evolution [51], the conservation of an amino acid on protein exposed surface [52]. The PSSM conservation score was used to quantify the conservation status of each amino acid in the protein sequence. Target sequences are scanned against the reference data sets UniRef100 (Release: 15.9, 13-Oct-2009) to generate the position specific scoring matrices (PSSMs) [41], [42], [43] using Position Specific Iterative BLAST (PSI BLAST) program (Release 2.2.12) [53].

In this study, Nearest Neighbor (NN) algorithm [54], [55], [56], [57] was used to construct classifiers to classify each sample to a fibril-forming one or a non-fibril-forming one. Besides the NN algorithm, random forest (RF) algorithm [33] was also used to construct classifier for it has been successfully applied in the diverse biological prediction problems [34], [35], [36]. RF classifier consists of many decision trees and makes decisions by choosing the class with the most votes of the decision trees in the forest.

Maximum Relevance, Minimum Redundancy method [58] is used to rank each feature according to both its relevance to the target (highly related to the prediction accuracy) and the redundancy between the features. A “good” feature is characterized by maximum relevance with the target variable and minimum redundancy within the features. With the mRMR result, we know the order of the features from the best feature to the worst feature. In order to get the optimal feature set which contains the optimal number of the features, Incremental Feature Selection (IFS) was used.

Jackknife Cross-Validation Method [54], [59] is used to evaluate statistical predictions. In Jackknife Cross-Validation Method, each sample in the data set is knocked out and tested by the predictor trained by the other samples in the data set.

To evaluate the performance of a predictor, the accurate rate for positive samples, negative samples and the overall accurate rate will be used:
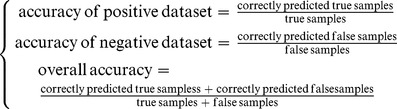



Please see the Text S1 for detailed description of the methods.

## Supporting Information

Table S1 **MaxRel feature list for amyloid prediction.**
(PDF)

Table S2 **mRMR feature list for amyloid prediction.**
(PDF)

Table S3 **The accuracies of the candidate models.**
(PDF)

Table S4 **The 446 features selected for feature analysis.**
(PDF)

Text S1 **The detailed description of the methods used in this research.**
(PDF)
