# Supplementary material for: Cooperativity among Short Amyloid Stretches in Long Amyloidogenic Sequences
Source: PLoS One. 2012 Jun 22;7(6):e39369. doi: 10.1371/journal.pone.0039369 (PMC3382238; doi:10.1371/journal.pone.0039369)
Supplement: Table S1 — MaxRel feature list for amyloid prediction. (PDF) [file pone.0039369.s001.pdf]

**Table S1. MaxRel feature list for amyloid prediction.**
Listed below are the 918 features prioritized by mRMR method according to Max-Relevance criterion. See the text of the paper for further explanation.

| Feature | | |
|---|---|---|
| Order | Amino Acid | Attribute |
| 1 | AA26 | Disorder |
| 2 | AA27 | Disorder |
| 3 | AA22 | Disorder |
| 4 | AA21 | Disorder |
| 5 | AA23 | Disorder |
| 6 | AA25 | Disorder |
| 7 | AA15 | Disorder |
| 8 | AA19 | Disorder |
| 9 | AA20 | Disorder |
| 10 | AA16 | Disorder |
| 11 | AA18 | Disorder |
| 12 | AA24 | Disorder |
| 13 | AA14 | Disorder |
| 14 | AA17 | Disorder |
| 15 | AA13 | Disorder |
| 16 | AA12 | Disorder |
| 17 | AA11 | Disorder |
| 18 | AA4 | Disorder |
| 19 | AA2 | Disorder |
| 20 | AA5 | Disorder |
| 21 | AA3 | Disorder |
| 22 | AA9 | Disorder |
| 23 | AA6 | Disorder |
| 24 | AA10 | Disorder |
| 25 | AA1 | Disorder |
| 26 | AA7 | Disorder |
| 27 | AA8 | Disorder |
| 28 | AA26 | Pssm_C |
| 29 | AA22 | Pssm_H |
| 30 | AA2 | Pssm_T |
| 31 | AA4 | Pssm_T |
| 32 | AA21 | Pssm_C |
| 33 | AA21 | Pssm_H |
| 34 | AA3 | Pssm_T |
| 35 | AA16 | Pssm_T |
| 36 | AA14 | Pssm_C |
| 37 | AA18 | Pssm_T |
| 38 | AA14 | Propensity of amino acid to be conserved at protein |
| 39 | AA25 | Pssm_H |
| 40 | AA11 | Pssm_C |
| 41 | AA23 | Pssm_H |
| 42 | AA13 | Pssm_C |

| | | |
|---|---|---|
| 43 | AA9 | Pssm_C |
| 44 | AA3 | Pssm_H |
| 45 | AA15 | Secondary Structure Strand |
| 46 | AA24 | Pssm_C |
| 47 | AA20 | Pssm_T |
| 48 | AA12 | Pssm_T |
| 49 | AA14 | Secondary Structure Strand |
| 50 | AA5 | Pssm_T |
| 51 | AA9 | Pssm_T |
| 52 | AA20 | Pssm_C |
| 53 | AA19 | Pssm_C |
| 54 | AA7 | Pssm_T |
| 55 | AA15 | Pssm_V |
| 56 | AA11 | Pssm_T |
| 57 | AA10 | Pssm_T |
| 58 | AA17 | Pssm_T |
| 59 | AA11 | Pssm_V |
| 60 | AA25 | Pssm_C |
| 61 | AA27 | Pssm_H |
| 62 | AA6 | Pssm_T |
| 63 | AA22 | Pssm_C |
| 64 | AA14 | Pssm_V |
| 65 | AA24 | Pssm_H |
| 66 | AA15 | Pssm_R |
| 67 | AA26 | Pssm_H |
| 68 | AA12 | Pssm_C |
| 69 | AA22 | Pssm_V |
| 70 | AA17 | Pssm_V |
| 71 | AA16 | Secondary Structure Strand |
| 72 | AA10 | Pssm_C |
| 73 | AA27 | Pssm_C |
| 74 | AA16 | Pssm_V |
| 75 | AA3 | Pssm_C |
| 76 | AA22 | Pssm_T |
| 77 | AA15 | Pssm_T |
| 78 | AA5 | Pssm_H |
| 79 | AA12 | Pssm_R |
| 80 | AA21 | Pssm_V |
| 81 | AA18 | Pssm_C |
| 82 | AA19 | Pssm_T |
| 83 | AA15 | Propensity of amino acid to be conserved at protein |
| 84 | AA17 | Propensity of amino acid to be conserved at protein |
| 85 | AA16 | Propensity of amino acid to be conserved at protein |
| 86 | AA12 | Propensity of amino acid to be conserved at protein |
| 87 | AA1 | Pssm_H |
| 88 | AA8 | Pssm_T |
| 89 | AA6 | Pssm_C |
| 90 | AA15 | Polarity |

| | | |
|---|---|---|
| 91 | AA13 | Pssm_T |
| 92 | AA12 | Pssm_V |
| 93 | AA7 | Pssm_C |
| 94 | AA3 | Pssm_Q |
| 95 | AA16 | Polarity |
| 96 | AA1 | Pssm_T |
| 97 | AA26 | Pssm_T |
| 98 | AA23 | Pssm_Q |
| 99 | AA23 | Pssm_V |
| 100 | AA14 | Pssm_T |
| 101 | AA13 | Propensity of amino acid to be conserved at protein |
| 102 | AA2 | Secondary Structure Helix |
| 103 | AA1 | Secondary Structure Helix |
| 104 | AA23 | Pssm_C |
| 105 | AA13 | Side Chain Count of Atom_C Deviation from Mean |
| 106 | AA2 | Pssm_C |
| 107 | AA19 | Pssm_D |
| 108 | AA23 | Pssm_T |
| 109 | AA2 | Pssm_R |
| 110 | AA13 | Secondary Structure Strand |
| 111 | AA13 | Pssm_V |
| 112 | AA4 | Pssm_D |
| 113 | AA21 | Pssm_T |
| 114 | AA22 | Pssm_Q |
| 115 | AA24 | Pssm_R |
| 116 | AA10 | Pssm_D |
| 117 | AA16 | Pssm_C |
| 118 | AA15 | Pssm_N |
| 119 | AA16 | Pssm_R |
| 120 | AA2 | Pssm_H |
| 121 | AA11 | Propensity of amino acid to be conserved at protein |
| 122 | AA12 | Pssm_K |
| 123 | AA15 | Pssm_H |
| 124 | AA5 | Pssm_C |
| 125 | AA3 | Secondary Structure Helix |
| 126 | AA19 | Propensity of amino acid to be conserved at protein |
| 127 | AA16 | Pssm_H |
| 128 | AA3 | Propensity of amino acid to be conserved at protein |
| 129 | AA2 | Propensity of amino acid to be conserved at protein |
| 130 | AA14 | Pssm_K |
| 131 | AA24 | Pssm_T |
| 132 | AA20 | Pssm_D |
| 133 | AA17 | Molecular Volume |
| 134 | AA10 | Pssm_H |
| 135 | AA4 | Pssm_Y |
| 136 | AA14 | Pssm_R |
| 137 | AA18 | Pssm_D |
| 138 | AA12 | Polarity |

| | | |
|---|---|---|
| 139 | AA10 | Polarity |
| 140 | AA26 | Solvent Accessibility Buried |
| 141 | AA4 | Secondary Structure Helix |
| 142 | AA15 | Pssm_Q |
| 143 | AA13 | Pssm_R |
| 144 | AA24 | Pssm_V |
| 145 | AA9 | Propensity of amino acid to be conserved at protein |
| 146 | AA15 | Gain/loss of amino acids during evolution |
| 147 | AA19 | Pssm_R |
| 148 | AA14 | Polarity |
| 149 | AA5 | Secondary Structure Helix |
| 150 | AA22 | Propensity of amino acid to be conserved at protein |
| 151 | AA20 | Pssm_H |
| 152 | AA27 | Gain/loss of amino acids during evolution |
| 153 | AA6 | Pssm_Q |
| 154 | AA17 | Pssm_H |
| 155 | AA19 | Polarity |
| 156 | AA12 | Pssm_Q |
| 157 | AA18 | Secondary Structure Strand |
| 158 | AA27 | Solvent Accessibility Buried |
| 159 | AA2 | Pssm_Q |
| 160 | AA3 | Pssm_V |
| 161 | AA25 | Pssm_T |
| 162 | AA27 | Pssm_V |
| 163 | AA21 | Pssm_R |
| 164 | AA13 | Pssm_K |
| 165 | AA4 | Propensity of amino acid to be conserved at protein |
| 166 | AA14 | Molecular Volume |
| 167 | AA7 | Pssm_Y |
| 168 | AA13 | Polarity |
| 169 | AA11 | Pssm_Y |
| 170 | AA7 | Pssm_R |
| 171 | AA10 | Pssm_R |
| 172 | AA1 | Pssm_Y |
| 173 | AA15 | Pssm_K |
| 174 | AA15 | Pssm_I |
| 175 | AA9 | Pssm_H |
| 176 | AA7 | Pssm_Q |
| 177 | AA18 | Pssm_H |
| 178 | AA8 | Pssm_Y |
| 179 | AA19 | Pssm_H |
| 180 | AA18 | Molecular Volume |
| 181 | AA2 | Pssm_L |
| 182 | AA18 | Pssm_V |
| 183 | AA21 | Propensity of amino acid to be conserved at protein |
| 184 | AA17 | Pssm_C |
| 185 | AA19 | Pssm_W |
| 186 | AA25 | Solvent Accessibility Buried |

| | | |
|---|---|---|
| 187 | AA15 | Secondary Structure Other |
| 188 | AA10 | Pssm_V |
| 189 | AA13 | Pssm_Y |
| 190 | AA12 | Molecular Volume |
| 191 | AA16 | Molecular Volume |
| 192 | AA5 | Propensity of amino acid to be conserved at protein |
| 193 | AA8 | Pssm_V |
| 194 | AA17 | Secondary Structure Strand |
| 195 | AA19 | Pssm_V |
| 196 | AA13 | Gain/loss of amino acids during evolution |
| 197 | AA12 | Side Chain Count of Atom_C Deviation from Mean |
| 198 | AA6 | Pssm_L |
| 199 | AA15 | Pssm_D |
| 200 | AA26 | Gain/loss of amino acids during evolution |
| 201 | AA15 | Pssm_C |
| 202 | AA6 | Pssm_D |
| 203 | AA1 | Pssm_V |
| 204 | AA4 | Pssm_C |
| 205 | AA14 | Pssm_H |
| 206 | AA10 | Propensity of amino acid to be conserved at protein |
| 207 | AA10 | Pssm_Y |
| 208 | AA25 | Pssm_Q |
| 209 | AA12 | Secondary Structure Strand |
| 210 | AA25 | Pssm_V |
| 211 | AA4 | Pssm_W |
| 212 | AA17 | Polarity |
| 213 | AA21 | Pssm_Y |
| 214 | AA6 | Secondary Structure Helix |
| 215 | AA18 | Propensity of amino acid to be conserved at protein |
| 216 | AA3 | Molecular Volume |
| 217 | AA12 | Pssm_N |
| 218 | AA12 | Pssm_H |
| 219 | AA21 | Pssm_W |
| 220 | AA27 | Pssm_P |
| 221 | AA14 | Electrostatic Charge |
| 222 | AA4 | Pssm_N |
| 223 | AA25 | Pssm_Y |
| 224 | AA13 | Pssm_H |
| 225 | AA24 | Pssm_D |
| 226 | AA26 | Polarity |
| 227 | AA22 | Polarity |
| 228 | AA20 | Pssm_V |
| 229 | AA16 | Pssm_K |
| 230 | AA3 | Pssm_R |
| 231 | AA27 | Pssm_T |
| 232 | AA24 | Gain/loss of amino acids during evolution |
| 233 | AA6 | Propensity of amino acid to be conserved at protein |
| 234 | AA8 | Propensity of amino acid to be conserved at protein |

| | | |
|---|---|---|
| 235 | AA10 | Pssm_K |
| 236 | AA1 | Pssm_W |
| 237 | AA16 | Secondary Structure Other |
| 238 | AA21 | Pssm_D |
| 239 | AA9 | Pssm_Y |
| 240 | AA3 | Pssm_W |
| 241 | AA13 | Pssm_Q |
| 242 | AA26 | Pssm_F |
| 243 | AA1 | Pssm_C |
| 244 | AA14 | Pssm_S |
| 245 | AA14 | Secondary Structure Other |
| 246 | AA26 | Pssm_P |
| 247 | AA24 | Pssm_K |
| 248 | AA11 | Polarity |
| 249 | AA6 | Pssm_E |
| 250 | AA14 | Pssm_E |
| 251 | AA20 | Pssm_W |
| 252 | AA11 | Pssm_R |
| 253 | AA23 | Pssm_R |
| 254 | AA9 | Pssm_W |
| 255 | AA13 | Molecular Volume |
| 256 | AA18 | Gain/loss of amino acids during evolution |
| 257 | AA14 | Pssm_N |
| 258 | AA4 | Pssm_H |
| 259 | AA5 | Pssm_R |
| 260 | AA17 | Pssm_D |
| 261 | AA19 | Secondary Structure Strand |
| 262 | AA17 | Pssm_R |
| 263 | AA4 | Secondary Structure Strand |
| 264 | AA3 | Secondary Structure Strand |
| 265 | AA20 | Polarity |
| 266 | AA8 | Pssm_C |
| 267 | AA15 | Pssm_E |
| 268 | AA27 | Pssm_L |
| 269 | AA7 | Pssm_K |
| 270 | AA22 | Pssm_W |
| 271 | AA2 | Polarity |
| 272 | AA18 | Pssm_W |
| 273 | AA14 | Pssm_Q |
| 274 | AA16 | Pssm_W |
| 275 | AA7 | Pssm_V |
| 276 | AA20 | Pssm_R |
| 277 | AA24 | Polarity |
| 278 | AA13 | Pssm_E |
| 279 | AA2 | Pssm_D |
| 280 | AA4 | Pssm_S |
| 281 | AA16 | Pssm_N |
| 282 | AA2 | Pssm_A |

| | | |
|---|---|---|
| 283 | AA22 | Pssm_R |
| 284 | AA4 | Pssm_R |
| 285 | AA15 | Pssm_W |
| 286 | AA7 | Polarity |
| 287 | AA4 | Pssm_A |
| 288 | AA5 | Pssm_Q |
| 289 | AA24 | Solvent Accessibility Buried |
| 290 | AA15 | Pssm_Y |
| 291 | AA6 | Pssm_Y |
| 292 | AA1 | Pssm_Q |
| 293 | AA10 | Pssm_N |
| 294 | AA3 | Pssm_A |
| 295 | AA10 | Pssm_W |
| 296 | AA5 | Pssm_K |
| 297 | AA1 | Pssm_A |
| 298 | AA15 | Pssm_S |
| 299 | AA15 | Molecular Volume |
| 300 | AA16 | Electrostatic Charge |
| 301 | AA9 | Pssm_P |
| 302 | AA17 | Pssm_W |
| 303 | AA2 | Pssm_P |
| 304 | AA10 | Pssm_E |
| 305 | AA9 | Pssm_V |
| 306 | AA24 | Pssm_Q |
| 307 | AA3 | Pssm_E |
| 308 | AA20 | Secondary Structure Strand |
| 309 | AA8 | Pssm_E |
| 310 | AA22 | Pssm_D |
| 311 | AA6 | Secondary Structure Strand |
| 312 | AA7 | Propensity of amino acid to be conserved at protein |
| 313 | AA7 | Pssm_A |
| 314 | AA27 | Pssm_Y |
| 315 | AA7 | Pssm_H |
| 316 | AA20 | Propensity of amino acid to be conserved at protein |
| 317 | AA11 | Pssm_W |
| 318 | AA5 | Secondary Structure Strand |
| 319 | AA22 | Pssm_I |
| 320 | AA12 | Pssm_D |
| 321 | AA6 | Molecular Volume |
| 322 | AA20 | Molecular Volume |
| 323 | AA4 | Pssm_Q |
| 324 | AA26 | Pssm_R |
| 325 | AA7 | Pssm_D |
| 326 | AA27 | Pssm_R |
| 327 | AA24 | Pssm_W |
| 328 | AA14 | Pssm_W |
| 329 | AA8 | Pssm_D |
| 330 | AA11 | Pssm_K |

| | | |
|---|---|---|
| 331 | AA22 | Pssm_Y |
| 332 | AA5 | Pssm_D |
| 333 | AA7 | Pssm_P |
| 334 | AA19 | Molecular Volume |
| 335 | AA14 | Pssm_D |
| 336 | AA16 | Gain/loss of amino acids during evolution |
| 337 | AA7 | Pssm_N |
| 338 | AA18 | Pssm_R |
| 339 | AA24 | Pssm_Y |
| 340 | AA7 | Secondary Structure Helix |
| 341 | AA1 | Secondary Structure Strand |
| 342 | AA24 | Pssm_A |
| 343 | AA18 | Polarity |
| 344 | AA5 | Pssm_N |
| 345 | AA6 | Pssm_G |
| 346 | AA23 | Pssm_D |
| 347 | AA4 | Pssm_P |
| 348 | AA14 | Pssm_I |
| 349 | AA23 | Pssm_Y |
| 350 | AA11 | Pssm_D |
| 351 | AA25 | Propensity of amino acid to be conserved at protein |
| 352 | AA21 | Pssm_Q |
| 353 | AA11 | Gain/loss of amino acids during evolution |
| 354 | AA2 | Molecular Volume |
| 355 | AA16 | Pssm_Q |
| 356 | AA6 | Pssm_N |
| 357 | AA3 | Pssm_Y |
| 358 | AA11 | Secondary Structure Strand |
| 359 | AA4 | Polarity |
| 360 | AA23 | Pssm_K |
| 361 | AA26 | Pssm_Y |
| 362 | AA15 | Electrostatic Charge |
| 363 | AA21 | Gain/loss of amino acids during evolution |
| 364 | AA2 | Pssm_W |
| 365 | AA10 | Pssm_Q |
| 366 | AA17 | Electrostatic Charge |
| 367 | AA13 | Electrostatic Charge |
| 368 | AA23 | Polarity |
| 369 | AA2 | Pssm_Y |
| 370 | AA16 | Pssm_D |
| 371 | AA19 | Pssm_Y |
| 372 | AA11 | Pssm_I |
| 373 | AA1 | Gain/loss of amino acids during evolution |
| 374 | AA16 | Pssm_L |
| 375 | AA3 | Pssm_N |
| 376 | AA19 | Pssm_N |
| 377 | AA13 | Pssm_A |
| 378 | AA13 | Pssm_W |

| | | |
|---|---|---|
| 379 | AA17 | Pssm_N |
| 380 | AA8 | Polarity |
| 381 | AA25 | Pssm_D |
| 382 | AA10 | Side Chain Count of Atom_C Deviation from Mean |
| 383 | AA4 | Pssm_E |
| 384 | AA8 | Secondary Structure Helix |
| 385 | AA5 | Polarity |
| 386 | AA6 | Pssm_P |
| 387 | AA10 | Gain/loss of amino acids during evolution |
| 388 | AA14 | Pssm_Y |
| 389 | AA8 | Pssm_A |
| 390 | AA2 | Pssm_V |
| 391 | AA5 | Pssm_A |
| 392 | AA25 | Gain/loss of amino acids during evolution |
| 393 | AA5 | Pssm_V |
| 394 | AA12 | Solvent Accessibility Buried |
| 395 | AA18 | Pssm_Q |
| 396 | AA27 | Pssm_Q |
| 397 | AA1 | Pssm_D |
| 398 | AA27 | Pssm_D |
| 399 | AA17 | Pssm_Y |
| 400 | AA26 | Pssm_Q |
| 401 | AA16 | Pssm_I |
| 402 | AA26 | Pssm_V |
| 403 | AA25 | Pssm_N |
| 404 | AA27 | Side Chain Count of Atom_C Deviation from Mean |
| 405 | AA5 | Pssm_Y |
| 406 | AA20 | Gain/loss of amino acids during evolution |
| 407 | AA6 | Pssm_V |
| 408 | AA8 | Pssm_F |
| 409 | AA19 | Pssm_Q |
| 410 | AA11 | Solvent Accessibility Buried |
| 411 | AA13 | Solvent Accessibility Buried |
| 412 | AA27 | Pssm_I |
| 413 | AA23 | Solvent Accessibility Buried |
| 414 | AA18 | Pssm_P |
| 415 | AA25 | Pssm_L |
| 416 | AA12 | Gain/loss of amino acids during evolution |
| 417 | AA11 | Pssm_L |
| 418 | AA24 | Pssm_N |
| 419 | AA1 | Propensity of amino acid to be conserved at protein |
| 420 | AA12 | Pssm_S |
| 421 | AA2 | Secondary Structure Strand |
| 422 | AA22 | Secondary Structure Other |
| 423 | AA7 | Molecular Volume |
| 424 | AA9 | Pssm_A |
| 425 | AA12 | Pssm_E |
| 426 | AA5 | Molecular Volume |

| | | |
|---|---|---|
| 427 | AA26 | Molecular Volume |
| 428 | AA18 | Secondary Structure Other |
| 429 | AA1 | Pssm_M |
| 430 | AA19 | Secondary Structure Other |
| 431 | AA12 | Electrostatic Charge |
| 432 | AA13 | Solvent Accessibility Exposed |
| 433 | AA12 | Pssm_Y |
| 434 | AA9 | Polarity |
| 435 | AA27 | Pssm_F |
| 436 | AA24 | Pssm_G |
| 437 | AA11 | Pssm_H |
| 438 | AA6 | Pssm_W |
| 439 | AA9 | Pssm_K |
| 440 | AA15 | Side Chain Count of Atom_C Deviation from Mean |
| 441 | AA12 | Solvent Accessibility Exposed |
| 442 | AA10 | Solvent Accessibility Buried |
| 443 | AA5 | Pssm_P |
| 444 | AA25 | Molecular Volume |
| 445 | AA1 | Molecular Volume |
| 446 | AA1 | Pssm_R |
| 447 | AA23 | Gain/loss of amino acids during evolution |
| 448 | AA21 | Pssm_E |
| 449 | AA26 | Pssm_E |
| 450 | AA5 | Pssm_L |
| 451 | AA9 | Pssm_N |
| 452 | AA27 | Pssm_A |
| 453 | AA7 | Pssm_L |
| 454 | AA25 | Pssm_P |
| 455 | AA22 | Molecular Volume |
| 456 | AA9 | Pssm_D |
| 457 | AA17 | Pssm_K |
| 458 | AA20 | Pssm_E |
| 459 | AA2 | Side Chain Count of Atom_C Deviation from Mean |
| 460 | AA14 | Pssm_L |
| 461 | AA15 | Solvent Accessibility Exposed |
| 462 | AA11 | Solvent Accessibility Exposed |
| 463 | AA27 | Polarity |
| 464 | AA26 | Pssm_L |
| 465 | AA22 | Pssm_P |
| 466 | AA13 | Secondary Structure Other |
| 467 | AA24 | Pssm_P |
| 468 | AA23 | Secondary Structure Other |
| 469 | AA23 | Pssm_W |
| 470 | AA1 | Pssm_S |
| 471 | AA9 | Pssm_R |
| 472 | AA26 | Propensity of amino acid to be conserved at protein |
| 473 | AA24 | Pssm_M |
| 474 | AA1 | Pssm_P |

| | | |
|---|---|---|
| 475 | AA17 | Secondary Structure Other |
| 476 | AA4 | Molecular Volume |
| 477 | AA25 | Pssm_W |
| 478 | AA17 | Side Chain Count of Atom_C Deviation from Mean |
| 479 | AA22 | Secondary Structure Strand |
| 480 | AA17 | Pssm_E |
| 481 | AA18 | Pssm_Y |
| 482 | AA8 | Pssm_H |
| 483 | AA25 | Pssm_E |
| 484 | AA19 | Gain/loss of amino acids during evolution |
| 485 | AA25 | Pssm_R |
| 486 | AA27 | Pssm_E |
| 487 | AA21 | Pssm_P |
| 488 | AA14 | Gain/loss of amino acids during evolution |
| 489 | AA8 | Pssm_P |
| 490 | AA17 | Gain/loss of amino acids during evolution |
| 491 | AA8 | Pssm_Q |
| 492 | AA3 | Pssm_D |
| 493 | AA24 | Pssm_F |
| 494 | AA2 | Pssm_G |
| 495 | AA6 | Pssm_H |
| 496 | AA27 | Pssm_N |
| 497 | AA10 | Molecular Volume |
| 498 | AA17 | Pssm_Q |
| 499 | AA4 | Pssm_V |
| 500 | AA8 | Pssm_R |
| 501 | AA26 | Side Chain Count of Atom_C Deviation from Mean |
| 502 | AA16 | Pssm_S |
| 503 | AA11 | Pssm_S |
| 504 | AA15 | Solvent Accessibility Buried |
| 505 | AA14 | Solvent Accessibility Buried |
| 506 | AA14 | Solvent Accessibility Exposed |
| 507 | AA21 | Secondary Structure Strand |
| 508 | AA3 | Polarity |
| 509 | AA9 | Molecular Volume |
| 510 | AA11 | Pssm_Q |
| 511 | AA24 | Propensity of amino acid to be conserved at protein |
| 512 | AA20 | Pssm_Q |
| 513 | AA18 | Pssm_F |
| 514 | AA22 | Gain/loss of amino acids during evolution |
| 515 | AA3 | Electrostatic Charge |
| 516 | AA6 | Pssm_A |
| 517 | AA27 | Secondary Structure |
| 518 | AA1 | Pssm_N |
| 519 | AA14 | Side Chain Count of Atom_C Deviation from Mean |
| 520 | AA12 | Pssm_I |
| 521 | AA10 | Solvent Accessibility Exposed |
| 522 | AA4 | Pssm_M |

| | | |
|---|---|---|
| 523 | AA12 | Pssm_A |
| 524 | AA7 | Pssm_E |
| 525 | AA22 | Solvent Accessibility Buried |
| 526 | AA4 | Electrostatic Charge |
| 527 | AA16 | Pssm_E |
| 528 | AA19 | Electrostatic Charge |
| 529 | AA5 | Pssm_E |
| 530 | AA23 | Molecular Volume |
| 531 | AA19 | Pssm_P |
| 532 | AA25 | Pssm_I |
| 533 | AA16 | Pssm_Y |
| 534 | AA9 | Pssm_E |
| 535 | AA22 | Pssm_N |
| 536 | AA22 | Pssm_S |
| 537 | AA21 | Secondary Structure Other |
| 538 | AA26 | Pssm_D |
| 539 | AA26 | Pssm_A |
| 540 | AA10 | Pssm_L |
| 541 | AA2 | Pssm_K |
| 542 | AA2 | Electrostatic Charge |
| 543 | AA23 | Pssm_N |
| 544 | AA8 | Pssm_W |
| 545 | AA21 | Polarity |
| 546 | AA13 | Pssm_L |
| 547 | AA23 | Secondary Structure Strand |
| 548 | AA15 | Pssm_L |
| 549 | AA9 | Pssm_Q |
| 550 | AA6 | Electrostatic Charge |
| 551 | AA18 | Electrostatic Charge |
| 552 | AA16 | Pssm_A |
| 553 | AA8 | Pssm_K |
| 554 | AA21 | Pssm_L |
| 555 | AA21 | Pssm_N |
| 556 | AA27 | Pssm_G |
| 557 | AA5 | Gain/loss of amino acids during evolution |
| 558 | AA7 | Secondary Structure Strand |
| 559 | AA4 | Gain/loss of amino acids during evolution |
| 560 | AA27 | Molecular Volume |
| 561 | AA19 | Pssm_E |
| 562 | AA5 | Side Chain Count of Atom_C Deviation from Mean |
| 563 | AA24 | Pssm_L |
| 564 | AA21 | Molecular Volume |
| 565 | AA10 | Pssm_G |
| 566 | AA11 | Pssm_E |
| 567 | AA22 | Pssm_F |
| 568 | AA5 | Pssm_M |
| 569 | AA4 | Pssm_K |
| 570 | AA14 | Pssm_P |

| | | |
|---|---|---|
| 571 | AA8 | Secondary Structure Other |
| 572 | AA9 | Pssm_G |
| 573 | AA3 | Pssm_M |
| 574 | AA20 | Secondary Structure Other |
| 575 | AA21 | Pssm_F |
| 576 | AA2 | Secondary Structure Other |
| 577 | AA23 | Pssm_S |
| 578 | AA8 | Pssm_L |
| 579 | AA2 | Pssm_E |
| 580 | AA9 | Secondary Structure Strand |
| 581 | AA9 | Gain/loss of amino acids during evolution |
| 582 | AA11 | Side Chain Count of Atom_C Deviation from Mean |
| 583 | AA19 | Pssm_I |
| 584 | AA1 | Codon Diversity |
| 585 | AA26 | Pssm_M |
| 586 | AA3 | Codon Diversity |
| 587 | AA11 | Molecular Volume |
| 588 | AA25 | Secondary Structure |
| 589 | AA17 | Pssm_M |
| 590 | AA3 | Pssm_L |
| 591 | AA13 | Pssm_F |
| 592 | AA26 | Solvent Accessibility Exposed |
| 593 | AA6 | Pssm_R |
| 594 | AA3 | Pssm_I |
| 595 | AA6 | Polarity |
| 596 | AA8 | Molecular Volume |
| 597 | AA20 | Pssm_I |
| 598 | AA9 | Secondary Structure |
| 599 | AA8 | Pssm_N |
| 600 | AA21 | Solvent Accessibility Buried |
| 601 | AA24 | Molecular Volume |
| 602 | AA10 | Secondary Structure Strand |
| 603 | AA4 | Side Chain Count of Atom_C Deviation from Mean |
| 604 | AA11 | Pssm_N |
| 605 | AA10 | Pssm_P |
| 606 | AA16 | Side Chain Count of Atom_C Deviation from Mean |
| 607 | AA12 | Pssm_L |
| 608 | AA23 | Pssm_I |
| 609 | AA19 | Pssm_A |
| 610 | AA7 | Side Chain Count of Atom_C Deviation from Mean |
| 611 | AA7 | Gain/loss of amino acids during evolution |
| 612 | AA6 | Pssm_S |
| 613 | AA8 | Solvent Accessibility Buried |
| 614 | AA23 | Pssm_M |
| 615 | AA13 | Pssm_N |
| 616 | AA9 | Codon Diversity |
| 617 | AA24 | Pssm_E |
| 618 | AA3 | Side Chain Count of Atom_C Deviation from Mean |

| | | |
|---|---|---|
| 619 | AA18 | Pssm_E |
| 620 | AA5 | Pssm_W |
| 621 | AA25 | Polarity |
| 622 | AA14 | Codon Diversity |
| 623 | AA20 | Pssm_A |
| 624 | AA20 | Pssm_Y |
| 625 | AA23 | Pssm_F |
| 626 | AA12 | Pssm_W |
| 627 | AA14 | Pssm_A |
| 628 | AA9 | Pssm_L |
| 629 | AA20 | Pssm_L |
| 630 | AA20 | Pssm_K |
| 631 | AA23 | Pssm_L |
| 632 | AA7 | Pssm_W |
| 633 | AA18 | Pssm_N |
| 634 | AA20 | Pssm_N |
| 635 | AA11 | Electrostatic Charge |
| 636 | AA25 | Pssm_A |
| 637 | AA12 | Pssm_M |
| 638 | AA23 | Electrostatic Charge |
| 639 | AA10 | Pssm_I |
| 640 | AA18 | Pssm_A |
| 641 | AA4 | Pssm_G |
| 642 | AA3 | Pssm_G |
| 643 | AA12 | Secondary Structure Other |
| 644 | AA9 | Electrostatic Charge |
| 645 | AA24 | Secondary Structure Other |
| 646 | AA15 | Secondary Structure |
| 647 | AA17 | Pssm_A |
| 648 | AA25 | Side Chain Count of Atom_C Deviation from Mean |
| 649 | AA9 | Secondary Structure Helix |
| 650 | AA21 | Codon Diversity |
| 651 | AA11 | Pssm_A |
| 652 | AA4 | Pssm_L |
| 653 | AA23 | Propensity of amino acid to be conserved at protein |
| 654 | AA6 | Pssm_K |
| 655 | AA18 | Secondary Structure |
| 656 | AA27 | Pssm_K |
| 657 | AA21 | Pssm_I |
| 658 | AA18 | Side Chain Count of Atom_C Deviation from Mean |
| 659 | AA24 | Side Chain Count of Atom_C Deviation from Mean |
| 660 | AA27 | Propensity of amino acid to be conserved at protein |
| 661 | AA21 | Pssm_G |
| 662 | AA25 | Solvent Accessibility Exposed |
| 663 | AA20 | Secondary Structure |
| 664 | AA9 | Pssm_I |
| 665 | AA7 | Secondary Structure Other |
| 666 | AA27 | Solvent Accessibility Exposed |

| | | |
|---|---|---|
| 667 | AA3 | Gain/loss of amino acids during evolution |
| 668 | AA1 | Pssm_E |
| 669 | AA3 | Pssm_K |
| 670 | AA1 | Pssm_L |
| 671 | AA10 | Electrostatic Charge |
| 672 | AA2 | Codon Diversity |
| 673 | AA11 | Codon Diversity |
| 674 | AA19 | Pssm_M |
| 675 | AA15 | Codon Diversity |
| 676 | AA5 | Pssm_S |
| 677 | AA10 | Pssm_A |
| 678 | AA14 | Pssm_M |
| 679 | AA16 | Codon Diversity |
| 680 | AA1 | Secondary Structure Other |
| 681 | AA7 | Solvent Accessibility Buried |
| 682 | AA6 | Side Chain Count of Atom_C Deviation from Mean |
| 683 | AA23 | Pssm_P |
| 684 | AA27 | Pssm_S |
| 685 | AA1 | Polarity |
| 686 | AA5 | Secondary Structure Other |
| 687 | AA17 | Pssm_S |
| 688 | AA25 | Electrostatic Charge |
| 689 | AA2 | Pssm_N |
| 690 | AA19 | Side Chain Count of Atom_C Deviation from Mean |
| 691 | AA13 | Pssm_D |
| 692 | AA2 | Gain/loss of amino acids during evolution |
| 693 | AA18 | Pssm_K |
| 694 | AA25 | Secondary Structure Other |
| 695 | AA20 | Solvent Accessibility Buried |
| 696 | AA9 | Solvent Accessibility Buried |
| 697 | AA8 | Solvent Accessibility Exposed |
| 698 | AA19 | Pssm_L |
| 699 | AA17 | Pssm_I |
| 700 | AA3 | Pssm_P |
| 701 | AA7 | Electrostatic Charge |
| 702 | AA21 | Electrostatic Charge |
| 703 | AA8 | Gain/loss of amino acids during evolution |
| 704 | AA17 | Pssm_P |
| 705 | AA2 | Secondary Structure |
| 706 | AA13 | Pssm_S |
| 707 | AA21 | Pssm_A |
| 708 | AA8 | Secondary Structure Strand |
| 709 | AA2 | Pssm_F |
| 710 | AA9 | Pssm_F |
| 711 | AA17 | Pssm_L |
| 712 | AA18 | Pssm_I |
| 713 | AA27 | Codon Diversity |
| 714 | AA3 | Pssm_S |

| | | |
|---|---|---|
| 715 | AA3 | Secondary Structure Other |
| 716 | AA26 | Pssm_K |
| 717 | AA10 | Pssm_S |
| 718 | AA22 | Pssm_L |
| 719 | AA26 | Secondary Structure Helix |
| 720 | AA27 | Secondary Structure Helix |
| 721 | AA26 | Secondary Structure Other |
| 722 | AA14 | Pssm_F |
| 723 | AA7 | Solvent Accessibility Exposed |
| 724 | AA6 | Codon Diversity |
| 725 | AA20 | Pssm_G |
| 726 | AA3 | Pssm_F |
| 727 | AA18 | Pssm_S |
| 728 | AA24 | Secondary Structure Strand |
| 729 | AA6 | Secondary Structure Other |
| 730 | AA4 | Secondary Structure Other |
| 731 | AA24 | Solvent Accessibility Exposed |
| 732 | AA16 | Solvent Accessibility Exposed |
| 733 | AA1 | Pssm_K |
| 734 | AA26 | Pssm_W |
| 735 | AA19 | Pssm_S |
| 736 | AA26 | Pssm_N |
| 737 | AA19 | Pssm_K |
| 738 | AA21 | Pssm_S |
| 739 | AA5 | Pssm_I |
| 740 | AA11 | Pssm_F |
| 741 | AA22 | Pssm_M |
| 742 | AA26 | Pssm_G |
| 743 | AA8 | Codon Diversity |
| 744 | AA22 | Electrostatic Charge |
| 745 | AA8 | Pssm_G |
| 746 | AA25 | Pssm_S |
| 747 | AA26 | Electrostatic Charge |
| 748 | AA5 | Pssm_G |
| 749 | AA23 | Secondary Structure |
| 750 | AA15 | Pssm_M |
| 751 | AA20 | Pssm_M |
| 752 | AA22 | Pssm_A |
| 753 | AA27 | Pssm_W |
| 754 | AA14 | Pssm_G |
| 755 | AA15 | Pssm_G |
| 756 | AA25 | Pssm_F |
| 757 | AA5 | Pssm_F |
| 758 | AA7 | Pssm_G |
| 759 | AA13 | Secondary Structure |
| 760 | AA24 | Secondary Structure |
| 761 | AA15 | Pssm_P |
| 762 | AA6 | Pssm_I |

| | | |
|---|---|---|
| 763 | AA25 | Secondary Structure Strand |
| 764 | AA7 | Pssm_I |
| 765 | AA27 | Secondary Structure Other |
| 766 | AA10 | Secondary Structure |
| 767 | AA9 | Solvent Accessibility Exposed |
| 768 | AA21 | Pssm_M |
| 769 | AA13 | Pssm_I |
| 770 | AA22 | Pssm_G |
| 771 | AA15 | Pssm_A |
| 772 | AA22 | Secondary Structure |
| 773 | AA25 | Secondary Structure Helix |
| 774 | AA26 | Secondary Structure Strand |
| 775 | AA11 | Pssm_P |
| 776 | AA2 | Pssm_S |
| 777 | AA16 | Solvent Accessibility Buried |
| 778 | AA13 | Pssm_P |
| 779 | AA26 | Secondary Structure |
| 780 | AA24 | Electrostatic Charge |
| 781 | AA26 | Pssm_I |
| 782 | AA19 | Codon Diversity |
| 783 | AA24 | Pssm_S |
| 784 | AA5 | Electrostatic Charge |
| 785 | AA20 | Secondary Structure Helix |
| 786 | AA8 | Pssm_M |
| 787 | AA5 | Codon Diversity |
| 788 | AA23 | Pssm_E |
| 789 | AA6 | Pssm_F |
| 790 | AA20 | Side Chain Count of Atom_C Deviation from Mean |
| 791 | AA16 | Pssm_F |
| 792 | AA7 | Codon Diversity |
| 793 | AA20 | Pssm_P |
| 794 | AA10 | Secondary Structure Helix |
| 795 | AA8 | Electrostatic Charge |
| 796 | AA6 | Gain/loss of amino acids during evolution |
| 797 | AA24 | Pssm_I |
| 798 | AA4 | Pssm_F |
| 799 | AA1 | Electrostatic Charge |
| 800 | AA16 | Secondary Structure |
| 801 | AA18 | Pssm_M |
| 802 | AA11 | Secondary Structure Other |
| 803 | AA16 | Pssm_M |
| 804 | AA23 | Solvent Accessibility Exposed |
| 805 | AA22 | Side Chain Count of Atom_C Deviation from Mean |
| 806 | AA12 | Pssm_G |
| 807 | AA12 | Pssm_P |
| 808 | AA26 | Pssm_S |
| 809 | AA19 | Solvent Accessibility Buried |
| 810 | AA2 | Pssm_I |

| | | |
|---|---|---|
| 811 | AA13 | Pssm_G |
| 812 | AA24 | Secondary Structure Helix |
| 813 | AA16 | Pssm_P |
| 814 | AA20 | Pssm_F |
| 815 | AA20 | Electrostatic Charge |
| 816 | AA1 | Side Chain Count of Atom_C Deviation from Mean |
| 817 | AA22 | Pssm_E |
| 818 | AA17 | Pssm_F |
| 819 | AA7 | Pssm_S |
| 820 | AA11 | Secondary Structure |
| 821 | AA1 | Pssm_F |
| 822 | AA2 | Pssm_M |
| 823 | AA17 | Solvent Accessibility Exposed |
| 824 | AA11 | Pssm_G |
| 825 | AA3 | Secondary Structure |
| 826 | AA19 | Pssm_F |
| 827 | AA11 | Secondary Structure Helix |
| 828 | AA9 | Side Chain Count of Atom_C Deviation from Mean |
| 829 | AA1 | Secondary Structure |
| 830 | AA25 | Codon Diversity |
| 831 | AA12 | Codon Diversity |
| 832 | AA25 | Pssm_G |
| 833 | AA23 | Side Chain Count of Atom_C Deviation from Mean |
| 834 | AA25 | Pssm_M |
| 835 | AA12 | Pssm_F |
| 836 | AA23 | Pssm_A |
| 837 | AA22 | Pssm_K |
| 838 | AA7 | Secondary Structure |
| 839 | AA8 | Side Chain Count of Atom_C Deviation from Mean |
| 840 | AA20 | Pssm_S |
| 841 | AA22 | Solvent Accessibility Exposed |
| 842 | AA15 | Pssm_F |
| 843 | AA17 | Codon Diversity |
| 844 | AA16 | Pssm_G |
| 845 | AA2 | Solvent Accessibility Buried |
| 846 | AA5 | Solvent Accessibility Exposed |
| 847 | AA21 | Secondary Structure |
| 848 | AA19 | Pssm_G |
| 849 | AA18 | Secondary Structure Helix |
| 850 | AA23 | Pssm_G |
| 851 | AA8 | Secondary Structure |
| 852 | AA7 | Pssm_M |
| 853 | AA21 | Side Chain Count of Atom_C Deviation from Mean |
| 854 | AA24 | Codon Diversity |
| 855 | AA27 | Secondary Structure Strand |
| 856 | AA9 | Pssm_M |
| 857 | AA8 | Pssm_I |
| 858 | AA6 | Pssm_M |

| | | |
|---|---|---|
| 859 | AA21 | Solvent Accessibility Exposed |
| 860 | AA21 | Secondary Structure Helix |
| 861 | AA18 | Pssm_L |
| 862 | AA22 | Codon Diversity |
| 863 | AA9 | Secondary Structure Other |
| 864 | AA5 | Solvent Accessibility Buried |
| 865 | AA17 | Solvent Accessibility Buried |
| 866 | AA20 | Solvent Accessibility Exposed |
| 867 | AA18 | Solvent Accessibility Exposed |
| 868 | AA25 | Pssm_K |
| 869 | AA23 | Secondary Structure Helix |
| 870 | AA4 | Codon Diversity |
| 871 | AA6 | Secondary Structure |
| 872 | AA17 | Secondary Structure |
| 873 | AA5 | Secondary Structure |
| 874 | AA26 | Codon Diversity |
| 875 | AA13 | Pssm_M |
| 876 | AA20 | Codon Diversity |
| 877 | AA11 | Pssm_M |
| 878 | AA21 | Pssm_K |
| 879 | AA14 | Secondary Structure Helix |
| 880 | AA12 | Secondary Structure Helix |
| 881 | AA4 | Pssm_I |
| 882 | AA18 | Codon Diversity |
| 883 | AA10 | Pssm_M |
| 884 | AA15 | Secondary Structure Helix |
| 885 | AA22 | Secondary Structure Helix |
| 886 | AA17 | Secondary Structure Helix |
| 887 | AA13 | Secondary Structure Helix |
| 888 | AA1 | Pssm_G |
| 889 | AA27 | Pssm_M |
| 890 | AA19 | Secondary Structure Helix |
| 891 | AA16 | Secondary Structure Helix |
| 892 | AA17 | Pssm_G |
| 893 | AA3 | Solvent Accessibility Exposed |
| 894 | AA10 | Codon Diversity |
| 895 | AA27 | Electrostatic Charge |
| 896 | AA7 | Pssm_F |
| 897 | AA9 | Pssm_S |
| 898 | AA2 | Solvent Accessibility Exposed |
| 899 | AA4 | Secondary Structure |
| 900 | AA8 | Pssm_S |
| 901 | AA19 | Solvent Accessibility Exposed |
| 902 | AA1 | Solvent Accessibility Exposed |
| 903 | AA14 | Secondary Structure |
| 904 | AA12 | Secondary Structure |
| 905 | AA18 | Pssm_G |
| 906 | AA23 | Codon Diversity |

| | | |
|---|---|---|
| 907 | AA10 | Pssm_F |
| 908 | AA1 | Pssm_I |
| 909 | AA13 | Codon Diversity |
| 910 | AA1 | Solvent Accessibility Buried |
| 911 | AA18 | Solvent Accessibility Buried |
| 912 | AA19 | Secondary Structure |
| 913 | AA4 | Solvent Accessibility Buried |
| 914 | AA3 | Solvent Accessibility Buried |
| 915 | AA6 | Solvent Accessibility Exposed |
| 916 | AA4 | Solvent Accessibility Exposed |
| 917 | AA6 | Solvent Accessibility Buried |
| 918 | AA10 | Secondary Structure Other |
