# Supplementary material for: Cooperativity among Short Amyloid Stretches in Long Amyloidogenic Sequences
Source: PLoS One. 2012 Jun 22;7(6):e39369. doi: 10.1371/journal.pone.0039369 (PMC3382238; doi:10.1371/journal.pone.0039369)
Supplement: Table S2 — mRMR feature list for amyloid prediction. (PDF) [file pone.0039369.s002.pdf]

Table S2. mRMR feature list for amyloid prediction.

Listed below are the 918 features prioritized by mRMR method according to both the Max-Relevance criterion and the Min-Redundancy criterion. In the table, the “AA26” represents the 26th amino acid residue of the peptide. See the text of the paper for further explanation.

Feature		
Order	Amino Acid	Attribute
1	AA26	Disorder
2	AA14	Propensity of amino acid to be conserved at protein
3	AA25	Pssm_H
4	AA14	Secondary Structure Strand
5	AA26	Pssm_C
6	AA11	Pssm_V
7	AA23	Pssm_H
8	AA16	Propensity of amino acid to be conserved at protein
9	AA13	Pssm_C
10	AA3	Pssm_H
11	AA21	Pssm_C
12	AA12	Propensity of amino acid to be conserved at protein
13	AA15	Propensity of amino acid to be conserved at protein
14	AA22	Pssm_H
15	AA17	Propensity of amino acid to be conserved at protein
16	AA24	Pssm_C
17	AA9	Pssm_C
18	AA2	Pssm_T
19	AA10	Polarity
20	AA13	Side Chain Count of Atom_C Deviation from Mean
21	AA20	Pssm_C
22	AA15	Pssm_V
23	AA14	Pssm_C
24	AA19	Propensity of amino acid to be conserved at protein
25	AA21	Pssm_H
26	AA27	Gain/loss of amino acids during evolution
27	AA4	Pssm_T
28	AA5	Propensity of amino acid to be conserved at protein
29	AA17	Pssm_V
30	AA22	Pssm_C
31	AA16	Pssm_V
32	AA18	Molecular Volume
33	AA11	Pssm_C
34	AA3	Propensity of amino acid to be conserved at protein
35	AA6	Pssm_C
36	AA27	Pssm_H
37	AA14	Pssm_V
38	AA15	Disorder
39	AA25	Pssm_C
40	AA9	Propensity of amino acid to be conserved at protein

41	AA24	Pssm_H
42	AA2	Propensity of amino acid to be conserved at protein
43	AA19	Pssm_C
44	AA11	Propensity of amino acid to be conserved at protein
45	AA5	Pssm_H
46	AA12	Pssm_V
47	AA26	Gain/loss of amino acids during evolution
48	AA3	Pssm_C
49	AA8	Propensity of amino acid to be conserved at protein
50	AA27	Pssm_C
51	AA3	Pssm_T
52	AA15	Gain/loss of amino acids during evolution
53	AA22	Pssm_V
54	AA16	Secondary Structure Strand
55	AA13	Propensity of amino acid to be conserved at protein
56	AA21	Propensity of amino acid to be conserved at protein
57	AA1	Pssm_H
58	AA6	Molecular Volume
59	AA12	Pssm_C
60	AA26	Pssm_H
61	AA24	Gain/loss of amino acids during evolution
62	AA16	Pssm_T
63	AA4	Propensity of amino acid to be conserved at protein
64	AA10	Pssm_C
65	AA7	Polarity
66	AA20	Molecular Volume
67	AA18	Pssm_C
68	AA21	Pssm_V
69	AA22	Propensity of amino acid to be conserved at protein
70	AA5	Pssm_T
71	AA7	Pssm_C
72	AA23	Pssm_V
73	AA2	Secondary Structure Helix
74	AA1	Gain/loss of amino acids during evolution
75	AA12	Pssm_R
76	AA4	Pssm_Y
77	AA26	Solvent Accessibility Buried
78	AA25	Gain/loss of amino acids during evolution
79	AA2	Pssm_C
80	AA18	Pssm_T
81	AA12	Side Chain Count of Atom_C Deviation from Mean
82	AA5	Pssm_C
83	AA13	Pssm_V
84	AA23	Pssm_C
85	AA17	Molecular Volume
86	AA7	Pssm_Y
87	AA9	Pssm_T
88	AA14	Molecular Volume

89	AA8	Pssm_Y
90	AA11	Gain/loss of amino acids during evolution
91	AA20	Secondary Structure Strand
92	AA15	Pssm_R
93	AA27	Solvent Accessibility Buried
94	AA13	Gain/loss of amino acids during evolution
95	AA27	Disorder
96	AA2	Pssm_R
97	AA1	Pssm_V
98	AA23	Gain/loss of amino acids during evolution
99	AA20	Pssm_T
100	AA15	Secondary Structure Strand
101	AA16	Gain/loss of amino acids during evolution
102	AA10	Pssm_H
103	AA21	Gain/loss of amino acids during evolution
104	AA24	Pssm_V
105	AA6	Secondary Structure Strand
106	AA2	Pssm_L
107	AA9	Pssm_Y
108	AA3	Molecular Volume
109	AA7	Pssm_T
110	AA18	Pssm_H
111	AA25	Solvent Accessibility Buried
112	AA18	Propensity of amino acid to be conserved at protein
113	AA17	Pssm_H
114	AA4	Pssm_C
115	AA27	Pssm_V
116	AA8	Pssm_V
117	AA12	Molecular Volume
118	AA16	Pssm_H
119	AA6	Propensity of amino acid to be conserved at protein
120	AA20	Polarity
121	AA19	Gain/loss of amino acids during evolution
122	AA10	Pssm_Y
123	AA11	Pssm_T
124	AA16	Pssm_C
125	AA1	Pssm_Y
126	AA4	Pssm_N
127	AA25	Pssm_Y
128	AA1	Secondary Structure Strand
129	AA3	Pssm_V
130	AA11	Pssm_Y
131	AA18	Gain/loss of amino acids during evolution
132	AA10	Pssm_T
133	AA19	Pssm_D
134	AA7	Molecular Volume
135	AA5	Gain/loss of amino acids during evolution
136	AA27	Side Chain Count of Atom_C Deviation from Mean

137	AA1	Pssm_C
138	AA6	Pssm_T
139	AA20	Pssm_H
140	AA8	Pssm_C
141	AA2	Molecular Volume
142	AA21	Disorder
143	AA11	Secondary Structure Strand
144	AA15	Pssm_H
145	AA10	Gain/loss of amino acids during evolution
146	AA23	Pssm_Q
147	AA16	Molecular Volume
148	AA18	Secondary Structure Strand
149	AA25	Propensity of amino acid to be conserved at protein
150	AA21	Pssm_Y
151	AA26	Molecular Volume
152	AA9	Gain/loss of amino acids during evolution
153	AA13	Pssm_Y
154	AA12	Pssm_T
155	AA22	Gain/loss of amino acids during evolution
156	AA19	Pssm_V
157	AA1	Propensity of amino acid to be conserved at protein
158	AA17	Side Chain Count of Atom_C Deviation from Mean
159	AA2	Pssm_H
160	AA6	Pssm_L
161	AA14	Pssm_S
162	AA4	Gain/loss of amino acids during evolution
163	AA24	Pssm_R
164	AA13	Molecular Volume
165	AA8	Gain/loss of amino acids during evolution
166	AA15	Side Chain Count of Atom_C Deviation from Mean
167	AA26	Pssm_Y
168	AA18	Pssm_V
169	AA2	Side Chain Count of Atom_C Deviation from Mean
170	AA17	Pssm_T
171	AA3	Pssm_Q
172	AA4	Secondary Structure Strand
173	AA9	Pssm_H
174	AA10	Side Chain Count of Atom_C Deviation from Mean
175	AA15	Pssm_C
176	AA6	Pssm_Y
177	AA25	Pssm_V
178	AA26	Polarity
179	AA13	Secondary Structure Strand
180	AA23	Pssm_Y
181	AA19	Molecular Volume
182	AA20	Gain/loss of amino acids during evolution
183	AA22	Pssm_T
184	AA12	Gain/loss of amino acids during evolution

185	AA15	Molecular Volume
186	AA14	Pssm_H
187	AA24	Pssm_Y
188	AA10	Pssm_V
189	AA23	Molecular Volume
190	AA14	Side Chain Count of Atom_C Deviation from Mean
191	AA5	Molecular Volume
192	AA24	Propensity of amino acid to be conserved at protein
193	AA3	Pssm_W
194	AA22	Secondary Structure Strand
195	AA1	Molecular Volume
196	AA13	Pssm_H
197	AA10	Propensity of amino acid to be conserved at protein
198	AA7	Pssm_R
199	AA27	Pssm_Y
200	AA2	Disorder
201	AA22	Pssm_Q
202	AA1	Pssm_A
203	AA16	Polarity
204	AA17	Gain/loss of amino acids during evolution
205	AA9	Pssm_V
206	AA27	Molecular Volume
207	AA19	Pssm_Y
208	AA5	Side Chain Count of Atom_C Deviation from Mean
209	AA17	Pssm_C
210	AA4	Molecular Volume
211	AA24	Side Chain Count of Atom_C Deviation from Mean
212	AA9	Molecular Volume
213	AA8	Pssm_T
214	AA25	Molecular Volume
215	AA19	Pssm_H
216	AA22	Molecular Volume
217	AA15	Polarity
218	AA20	Pssm_V
219	AA3	Side Chain Count of Atom_C Deviation from Mean
220	AA1	Pssm_M
221	AA12	Pssm_H
222	AA5	Pssm_V
223	AA24	Solvent Accessibility Buried
224	AA1	Secondary Structure Helix
225	AA11	Side Chain Count of Atom_C Deviation from Mean
226	AA21	Molecular Volume
227	AA18	Pssm_D
228	AA7	Pssm_V
229	AA4	Pssm_M
230	AA22	Pssm_Y
231	AA15	Pssm_T
232	AA14	Gain/loss of amino acids during evolution

233	AA1	Pssm_W
234	AA7	Gain/loss of amino acids during evolution
235	AA3	Pssm_A
236	AA24	Pssm_M
237	AA22	Disorder
238	AA4	Pssm_H
239	AA10	Pssm_D
240	AA2	Gain/loss of amino acids during evolution
241	AA26	Side Chain Count of Atom_C Deviation from Mean
242	AA11	Molecular Volume
243	AA7	Propensity of amino acid to be conserved at protein
244	AA23	Polarity
245	AA9	Secondary Structure Strand
246	AA6	Gain/loss of amino acids during evolution
247	AA8	Molecular Volume
248	AA23	Pssm_T
249	AA13	Pssm_R
250	AA20	Propensity of amino acid to be conserved at protein
251	AA25	Side Chain Count of Atom_C Deviation from Mean
252	AA5	Pssm_Y
253	AA11	Solvent Accessibility Buried
254	AA3	Secondary Structure Strand
255	AA19	Secondary Structure Strand
256	AA18	Side Chain Count of Atom_C Deviation from Mean
257	AA14	Polarity
258	AA15	Pssm_Y
259	AA26	Pssm_T
260	AA2	Pssm_A
261	AA19	Polarity
262	AA4	Pssm_A
263	AA6	Pssm_N
264	AA3	Pssm_Y
265	AA12	Polarity
266	AA17	Pssm_M
267	AA27	Secondary Structure Strand
268	AA21	Pssm_R
269	AA22	Polarity
270	AA2	Pssm_Y
271	AA16	Pssm_R
272	AA24	Molecular Volume
273	AA15	Pssm_N
274	AA10	Molecular Volume
275	AA26	Pssm_M
276	AA16	Side Chain Count of Atom_C Deviation from Mean
277	AA24	Pssm_A
278	AA4	Pssm_D
279	AA12	Secondary Structure Strand
280	AA14	Pssm_Y

281	AA7	Side Chain Count of Atom_C Deviation from Mean
282	AA27	Pssm_L
283	AA15	Secondary Structure Other
284	AA6	Side Chain Count of Atom_C Deviation from Mean
285	AA20	Pssm_D
286	AA1	Pssm_T
287	AA8	Pssm_H
288	AA12	Pssm_Y
289	AA4	Side Chain Count of Atom_C Deviation from Mean
290	AA27	Pssm_A
291	AA13	Pssm_T
292	AA3	Gain/loss of amino acids during evolution
293	AA5	Pssm_N
294	AA7	Pssm_H
295	AA17	Polarity
296	AA23	Secondary Structure Strand
297	AA11	Pssm_H
298	AA14	Electrostatic Charge
299	AA8	Side Chain Count of Atom_C Deviation from Mean
300	AA25	Disorder
301	AA2	Pssm_V
302	AA17	Secondary Structure Strand
303	AA20	Side Chain Count of Atom_C Deviation from Mean
304	AA17	Pssm_Y
305	AA23	Pssm_M
306	AA13	Polarity
307	AA19	Side Chain Count of Atom_C Deviation from Mean
308	AA21	Side Chain Count of Atom_C Deviation from Mean
309	AA3	Pssm_M
310	AA18	Pssm_Y
311	AA10	Pssm_R
312	AA11	Polarity
313	AA12	Pssm_M
314	AA14	Pssm_R
315	AA9	Side Chain Count of Atom_C Deviation from Mean
316	AA25	Pssm_Q
317	AA6	Pssm_V
318	AA19	Pssm_T
319	AA27	Propensity of amino acid to be conserved at protein
320	AA5	Secondary Structure Strand
321	AA26	Pssm_F
322	AA21	Pssm_M
323	AA12	Pssm_N
324	AA8	Pssm_A
325	AA5	Pssm_M
326	AA23	Solvent Accessibility Buried
327	AA21	Pssm_T
328	AA7	Pssm_A

329	AA16	Electrostatic Charge
330	AA24	Polarity
331	AA9	Pssm_N
332	AA8	Polarity
333	AA1	Pssm_N
334	AA22	Side Chain Count of Atom_C Deviation from Mean
335	AA14	Disorder
336	AA16	Pssm_Y
337	AA2	Polarity
338	AA17	Electrostatic Charge
339	AA3	Pssm_R
340	AA26	Propensity of amino acid to be conserved at protein
341	AA27	Pssm_R
342	AA5	Pssm_R
343	AA1	Side Chain Count of Atom_C Deviation from Mean
344	AA6	Pssm_H
345	AA25	Secondary Structure Strand
346	AA19	Pssm_R
347	AA13	Electrostatic Charge
348	AA23	Side Chain Count of Atom_C Deviation from Mean
349	AA15	Electrostatic Charge
350	AA18	Polarity
351	AA20	Pssm_M
352	AA5	Secondary Structure Helix
353	AA5	Pssm_A
354	AA25	Pssm_M
355	AA21	Codon Diversity
356	AA19	Pssm_M
357	AA9	Codon Diversity
358	AA27	Secondary Structure
359	AA14	Pssm_M
360	AA16	Pssm_M
361	AA8	Pssm_M
362	AA4	Polarity
363	AA26	Pssm_V
364	AA14	Pssm_T
365	AA2	Pssm_Q
366	AA1	Codon Diversity
367	AA15	Pssm_I
368	AA21	Secondary Structure Strand
369	AA20	Pssm_Y
370	AA13	Solvent Accessibility Buried
371	AA22	Pssm_M
372	AA7	Pssm_N
373	AA25	Secondary Structure
374	AA19	Disorder
375	AA3	Codon Diversity
376	AA7	Secondary Structure Strand

377	AA12	Pssm_K
378	AA23	Propensity of amino acid to be conserved at protein
379	AA6	Electrostatic Charge
380	AA13	Pssm_A
381	AA26	Pssm_R
382	AA5	Polarity
383	AA2	Secondary Structure Strand
384	AA11	Pssm_R
385	AA3	Pssm_N
386	AA9	Pssm_M
387	AA7	Pssm_M
388	AA27	Pssm_F
389	AA2	Pssm_M
390	AA18	Pssm_M
391	AA22	Solvent Accessibility Buried
392	AA10	Pssm_N
393	AA16	Pssm_N
394	AA24	Pssm_T
395	AA4	Pssm_S
396	AA12	Electrostatic Charge
397	AA22	Secondary Structure Other
398	AA11	Codon Diversity
399	AA21	Pssm_D
400	AA1	Pssm_S
401	AA26	Pssm_A
402	AA10	Pssm_M
403	AA18	Electrostatic Charge
404	AA26	Secondary Structure Strand
405	AA20	Pssm_R
406	AA12	Solvent Accessibility Buried
407	AA9	Secondary Structure
408	AA11	Pssm_M
409	AA25	Pssm_N
410	AA14	Pssm_K
411	AA6	Pssm_A
412	AA16	Pssm_W
413	AA27	Codon Diversity
414	AA8	Codon Diversity
415	AA2	Electrostatic Charge
416	AA14	Secondary Structure Other
417	AA10	Solvent Accessibility Buried
418	AA23	Pssm_R
419	AA13	Pssm_M
420	AA3	Electrostatic Charge
421	AA22	Pssm_F
422	AA4	Pssm_V
423	AA17	Pssm_R
424	AA25	Pssm_A

425	AA6	Codon Diversity
426	AA10	Secondary Structure Strand
427	AA19	Electrostatic Charge
428	AA18	Pssm_F
429	AA7	Codon Diversity
430	AA12	Pssm_A
431	AA23	Electrostatic Charge
432	AA15	Codon Diversity
433	AA4	Pssm_W
434	AA21	Solvent Accessibility Buried
435	AA8	Pssm_D
436	AA27	Pssm_M
437	AA6	Pssm_Q
438	AA24	Pssm_F
439	AA23	Disorder
440	AA16	Codon Diversity
441	AA22	Pssm_I
442	AA25	Codon Diversity
443	AA14	Codon Diversity
444	AA4	Electrostatic Charge
445	AA20	Codon Diversity
446	AA1	Solvent Accessibility Exposed
447	AA6	Pssm_M
448	AA3	Secondary Structure Helix
449	AA15	Pssm_M
450	AA9	Pssm_A
451	AA5	Codon Diversity
452	AA1	Secondary Structure
453	AA25	Pssm_T
454	AA24	Secondary Structure Strand
455	AA27	Pssm_T
456	AA9	Pssm_W
457	AA10	Electrostatic Charge
458	AA26	Codon Diversity
459	AA15	Pssm_S
460	AA17	Pssm_N
461	AA9	Electrostatic Charge
462	AA2	Codon Diversity
463	AA14	Pssm_N
464	AA22	Pssm_R
465	AA21	Electrostatic Charge
466	AA20	Pssm_W
467	AA7	Pssm_L
468	AA24	Electrostatic Charge
469	AA19	Secondary Structure Other
470	AA11	Electrostatic Charge
471	AA19	Codon Diversity
472	AA4	Pssm_R

473	AA8	Pssm_F
474	AA18	Codon Diversity
475	AA13	Pssm_K
476	AA7	Electrostatic Charge
477	AA24	Pssm_N
478	AA14	Pssm_W
479	AA8	Electrostatic Charge
480	AA25	Electrostatic Charge
481	AA11	Pssm_S
482	AA23	Secondary Structure
483	AA20	Pssm_A
484	AA17	Codon Diversity
485	AA16	Pssm_L
486	AA6	Pssm_D
487	AA22	Codon Diversity
488	AA1	Electrostatic Charge
489	AA5	Electrostatic Charge
490	AA3	Polarity
491	AA19	Pssm_W
492	AA8	Secondary Structure Strand
493	AA15	Solvent Accessibility Exposed
494	AA27	Pssm_Q
495	AA2	Secondary Structure
496	AA26	Electrostatic Charge
497	AA21	Pssm_F
498	AA9	Polarity
499	AA16	Secondary Structure Other
500	AA10	Pssm_A
501	AA7	Solvent Accessibility Exposed
502	AA5	Pssm_L
503	AA18	Secondary Structure
504	AA12	Codon Diversity
505	AA25	Pssm_L
506	AA20	Electrostatic Charge
507	AA24	Secondary Structure
508	AA16	Disorder
509	AA13	Codon Diversity
510	AA21	Secondary Structure
511	AA2	Pssm_D
512	AA19	Pssm_A
513	AA1	Pssm_R
514	AA23	Pssm_F
515	AA10	Codon Diversity
516	AA17	Pssm_W
517	AA26	Pssm_Q
518	AA27	Polarity
519	AA6	Secondary Structure
520	AA3	Solvent Accessibility Exposed

521	AA23	Codon Diversity
522	AA15	Secondary Structure
523	AA4	Codon Diversity
524	AA26	Secondary Structure
525	AA10	Pssm_W
526	AA22	Secondary Structure
527	AA11	Pssm_L
528	AA24	Pssm_D
529	AA14	Solvent Accessibility Buried
530	AA9	Solvent Accessibility Buried
531	AA18	Pssm_R
532	AA19	Pssm_F
533	AA16	Pssm_A
534	AA8	Pssm_R
535	AA5	Secondary Structure
536	AA23	Pssm_S
537	AA20	Secondary Structure
538	AA14	Pssm_A
539	AA11	Secondary Structure
540	AA26	Solvent Accessibility Exposed
541	AA7	Secondary Structure
542	AA13	Secondary Structure
543	AA12	Pssm_Q
544	AA10	Secondary Structure
545	AA5	Solvent Accessibility Exposed
546	AA24	Codon Diversity
547	AA7	Pssm_K
548	AA20	Solvent Accessibility Buried
549	AA6	Pssm_G
550	AA22	Electrostatic Charge
551	AA19	Pssm_N
552	AA17	Secondary Structure
553	AA27	Pssm_I
554	AA8	Secondary Structure
555	AA3	Secondary Structure
556	AA10	Pssm_K
557	AA15	Pssm_W
558	AA12	Secondary Structure
559	AA21	Polarity
560	AA15	Pssm_K
561	AA25	Solvent Accessibility Exposed
562	AA1	Pssm_Q
563	AA13	Pssm_F
564	AA18	Pssm_W
565	AA27	Electrostatic Charge
566	AA4	Secondary Structure Helix
567	AA11	Pssm_A
568	AA16	Pssm_K

569	AA21	Pssm_A
570	AA2	Solvent Accessibility Buried
571	AA24	Pssm_Q
572	AA22	Pssm_A
573	AA17	Pssm_D
574	AA8	Solvent Accessibility Buried
575	AA9	Pssm_R
576	AA14	Pssm_L
577	AA16	Pssm_F
578	AA26	Pssm_L
579	AA3	Pssm_I
580	AA19	Secondary Structure
581	AA25	Pssm_F
582	AA14	Secondary Structure
583	AA18	Pssm_A
584	AA16	Secondary Structure
585	AA27	Pssm_N
586	AA5	Pssm_K
587	AA25	Pssm_R
588	AA23	Secondary Structure Other
589	AA9	Pssm_F
590	AA4	Secondary Structure
591	AA12	Pssm_S
592	AA21	Pssm_W
593	AA2	Pssm_F
594	AA1	Polarity
595	AA11	Pssm_K
596	AA17	Pssm_A
597	AA23	Pssm_A
598	AA22	Pssm_S
599	AA1	Pssm_F
600	AA6	Polarity
601	AA7	Pssm_Q
602	AA27	Secondary Structure Other
603	AA23	Pssm_K
604	AA11	Pssm_W
605	AA4	Solvent Accessibility Exposed
606	AA13	Solvent Accessibility Exposed
607	AA2	Pssm_G
608	AA24	Pssm_K
609	AA12	Pssm_F
610	AA16	Pssm_I
611	AA25	Polarity
612	AA11	Pssm_I
613	AA27	Solvent Accessibility Exposed
614	AA21	Pssm_N
615	AA5	Pssm_D
616	AA8	Pssm_N

617	AA2	Pssm_W
618	AA14	Pssm_I
619	AA3	Pssm_F
620	AA20	Disorder
621	AA18	Pssm_N
622	AA24	Pssm_L
623	AA11	Solvent Accessibility Exposed
624	AA20	Pssm_F
625	AA16	Solvent Accessibility Exposed
626	AA1	Secondary Structure Other
627	AA15	Pssm_D
628	AA4	Pssm_L
629	AA10	Pssm_L
630	AA5	Pssm_F
631	AA22	Pssm_N
632	AA8	Secondary Structure Other
633	AA23	Pssm_N
634	AA3	Pssm_E
635	AA20	Pssm_N
636	AA1	Pssm_L
637	AA12	Pssm_D
638	AA15	Pssm_F
639	AA18	Solvent Accessibility Exposed
640	AA7	Pssm_D
641	AA25	Pssm_I
642	AA6	Pssm_F
643	AA14	Pssm_F
644	AA3	Pssm_L
645	AA9	Pssm_K
646	AA4	Pssm_F
647	AA17	Solvent Accessibility Exposed
648	AA6	Secondary Structure Helix
649	AA21	Pssm_L
650	AA16	Pssm_S
651	AA15	Pssm_Q
652	AA23	Pssm_L
653	AA10	Solvent Accessibility Exposed
654	AA2	Pssm_N
655	AA26	Pssm_N
656	AA8	Pssm_L
657	AA11	Pssm_N
658	AA18	Secondary Structure Other
659	AA12	Solvent Accessibility Exposed
660	AA27	Pssm_G
661	AA1	Pssm_D
662	AA13	Pssm_N
663	AA7	Pssm_F
664	AA20	Pssm_I

665	AA17	Pssm_F
666	AA22	Pssm_W
667	AA3	Pssm_S
668	AA24	Solvent Accessibility Exposed
669	AA9	Pssm_L
670	AA25	Pssm_D
671	AA19	Solvent Accessibility Buried
672	AA6	Pssm_W
673	AA13	Secondary Structure Other
674	AA19	Pssm_I
675	AA6	Solvent Accessibility Exposed
676	AA1	Solvent Accessibility Buried
677	AA15	Pssm_A
678	AA10	Pssm_F
679	AA24	Pssm_W
680	AA27	Pssm_S
681	AA13	Pssm_W
682	AA24	Pssm_G
683	AA22	Solvent Accessibility Exposed
684	AA14	Pssm_D
685	AA6	Pssm_S
686	AA3	Pssm_G
687	AA12	Pssm_I
688	AA26	Pssm_S
689	AA2	Pssm_I
690	AA21	Pssm_Q
691	AA20	Pssm_L
692	AA27	Secondary Structure Helix
693	AA9	Pssm_D
694	AA5	Pssm_S
695	AA7	Solvent Accessibility Buried
696	AA23	Pssm_I
697	AA11	Pssm_F
698	AA4	Pssm_Q
699	AA2	Pssm_K
700	AA14	Solvent Accessibility Exposed
701	AA23	Solvent Accessibility Exposed
702	AA6	Pssm_R
703	AA17	Pssm_S
704	AA15	Pssm_L
705	AA21	Pssm_I
706	AA3	Solvent Accessibility Buried
707	AA11	Pssm_D
708	AA19	Pssm_S
709	AA8	Pssm_K
710	AA25	Secondary Structure Other
711	AA1	Pssm_I
712	AA2	Secondary Structure Other

713	AA18	Pssm_L
714	AA25	Pssm_S
715	AA13	Pssm_L
716	AA26	Pssm_G
717	AA5	Pssm_I
718	AA10	Pssm_G
719	AA15	Solvent Accessibility Buried
720	AA21	Solvent Accessibility Exposed
721	AA22	Pssm_L
722	AA23	Pssm_W
723	AA5	Pssm_Q
724	AA27	Pssm_P
725	AA19	Pssm_L
726	AA2	Solvent Accessibility Exposed
727	AA9	Pssm_G
728	AA7	Pssm_I
729	AA21	Secondary Structure Other
730	AA17	Pssm_I
731	AA18	Pssm_S
732	AA13	Pssm_S
733	AA20	Pssm_G
734	AA12	Pssm_W
735	AA2	Pssm_S
736	AA4	Solvent Accessibility Buried
737	AA8	Pssm_G
738	AA20	Solvent Accessibility Exposed
739	AA22	Pssm_D
740	AA6	Pssm_I
741	AA12	Pssm_L
742	AA27	Pssm_D
743	AA21	Pssm_S
744	AA9	Solvent Accessibility Exposed
745	AA3	Pssm_D
746	AA24	Pssm_I
747	AA26	Pssm_I
748	AA7	Secondary Structure Helix
749	AA4	Pssm_G
750	AA1	Pssm_G
751	AA16	Pssm_D
752	AA10	Pssm_S
753	AA13	Pssm_Q
754	AA26	Pssm_K
755	AA8	Solvent Accessibility Exposed
756	AA9	Pssm_I
757	AA17	Pssm_L
758	AA23	Pssm_D
759	AA22	Pssm_G
760	AA7	Pssm_S

761	AA24	Disorder
762	AA8	Pssm_W
763	AA14	Pssm_Q
764	AA25	Pssm_G
765	AA17	Secondary Structure Other
766	AA5	Pssm_G
767	AA24	Pssm_S
768	AA4	Pssm_K
769	AA13	Pssm_I
770	AA10	Pssm_I
771	AA16	Solvent Accessibility Buried
772	AA18	Pssm_I
773	AA5	Pssm_W
774	AA8	Pssm_S
775	AA12	Secondary Structure Other
776	AA5	Solvent Accessibility Buried
777	AA27	Pssm_W
778	AA23	Pssm_G
779	AA20	Pssm_S
780	AA27	Pssm_E
781	AA4	Pssm_I
782	AA17	Pssm_K
783	AA26	Pssm_E
784	AA21	Pssm_G
785	AA7	Pssm_W
786	AA9	Pssm_S
787	AA18	Pssm_Q
788	AA12	Pssm_G
789	AA6	Pssm_E
790	AA1	Pssm_K
791	AA25	Pssm_W
792	AA19	Solvent Accessibility Exposed
793	AA11	Pssm_G
794	AA26	Secondary Structure Other
795	AA14	Pssm_G
796	AA3	Pssm_K
797	AA8	Pssm_Q
798	AA18	Solvent Accessibility Buried
799	AA7	Pssm_G
800	AA20	Pssm_Q
801	AA19	Pssm_G
802	AA27	Pssm_K
803	AA8	Pssm_I
804	AA7	Secondary Structure Other
805	AA16	Pssm_Q
806	AA10	Pssm_Q
807	AA20	Secondary Structure Other
808	AA17	Solvent Accessibility Buried

809	AA6	Solvent Accessibility Buried
810	AA15	Pssm_G
811	AA10	Secondary Structure Other
812	AA26	Pssm_P
813	AA13	Pssm_G
814	AA19	Pssm_Q
815	AA4	Secondary Structure Other
816	AA22	Pssm_K
817	AA24	Secondary Structure Other
818	AA17	Pssm_G
819	AA26	Pssm_D
820	AA8	Pssm_E
821	AA14	Pssm_E
822	AA16	Pssm_G
823	AA18	Pssm_G
824	AA25	Pssm_K
825	AA26	Secondary Structure Helix
826	AA20	Pssm_K
827	AA17	Pssm_Q
828	AA26	Pssm_W
829	AA2	Pssm_P
830	AA13	Pssm_E
831	AA8	Secondary Structure Helix
832	AA25	Pssm_E
833	AA9	Pssm_Q
834	AA2	Pssm_E
835	AA12	Pssm_E
836	AA6	Pssm_K
837	AA1	Disorder
838	AA11	Pssm_Q
839	AA10	Pssm_E
840	AA4	Pssm_E
841	AA3	Secondary Structure Other
842	AA21	Pssm_E
843	AA18	Pssm_K
844	AA13	Pssm_D
845	AA19	Pssm_K
846	AA11	Secondary Structure Other
847	AA1	Pssm_P
848	AA7	Pssm_E
849	AA24	Pssm_E
850	AA5	Secondary Structure Other
851	AA22	Pssm_E
852	AA15	Pssm_E
853	AA5	Pssm_E
854	AA20	Pssm_E
855	AA23	Pssm_E
856	AA21	Pssm_K

857	AA9	Secondary Structure Other
858	AA1	Pssm_E
859	AA7	Pssm_P
860	AA25	Secondary Structure Helix
861	AA4	Pssm_P
862	AA9	Pssm_E
863	AA25	Pssm_P
864	AA18	Pssm_E
865	AA6	Secondary Structure Other
866	AA16	Pssm_E
867	AA11	Disorder
868	AA11	Pssm_E
869	AA20	Secondary Structure Helix
870	AA17	Pssm_E
871	AA19	Pssm_E
872	AA14	Secondary Structure Helix
873	AA3	Pssm_P
874	AA6	Pssm_P
875	AA8	Pssm_P
876	AA24	Secondary Structure Helix
877	AA22	Pssm_P
878	AA4	Disorder
879	AA9	Secondary Structure Helix
880	AA18	Secondary Structure Helix
881	AA24	Pssm_P
882	AA21	Pssm_P
883	AA18	Pssm_P
884	AA9	Pssm_P
885	AA5	Pssm_P
886	AA17	Disorder
887	AA13	Secondary Structure Helix
888	AA23	Secondary Structure Helix
889	AA19	Pssm_P
890	AA14	Pssm_P
891	AA10	Pssm_P
892	AA21	Secondary Structure Helix
893	AA10	Secondary Structure Helix
894	AA11	Pssm_P
895	AA23	Pssm_P
896	AA16	Secondary Structure Helix
897	AA18	Disorder
898	AA19	Secondary Structure Helix
899	AA12	Pssm_P
900	AA15	Pssm_P
901	AA3	Disorder
902	AA12	Secondary Structure Helix
903	AA20	Pssm_P
904	AA22	Secondary Structure Helix

905	AA17	Pssm_P
906	AA13	Pssm_P
907	AA16	Pssm_P
908	AA11	Secondary Structure Helix
909	AA13	Disorder
910	AA17	Secondary Structure Helix
911	AA15	Secondary Structure Helix
912	AA12	Disorder
913	AA5	Disorder
914	AA6	Disorder
915	AA9	Disorder
916	AA7	Disorder
917	AA10	Disorder
918	AA8	Disorder
