# Supplementary material for: Cooperativity among Short Amyloid Stretches in Long Amyloidogenic Sequences
Source: PLoS One. 2012 Jun 22;7(6):e39369. doi: 10.1371/journal.pone.0039369 (PMC3382238; doi:10.1371/journal.pone.0039369)
Supplement: Table S3 — The accuracies of the candidate models. (PDF) [file pone.0039369.s003.pdf]

Table S3. The accuracies of the candidate models.

The highest accuracy of the model with all the 918 features reached 70.7%. And the accuracies listed in this table correspond to the curve with 27-residue peptide in the Figure 1A.

Candidate Model	Accuracy (%)	Candidate Model	Accuracy (%)	Candidate Model	Accuracy (%)	Candidate Model	Accuracy (%)
2	52.9	232	68.03	462	67.23	692	68.29
3	55.83	233	68.47	463	67.41	693	68.07
4	58.42	234	68.14	464	67.59	694	67.96
5	54.4	235	68.4	465	67.48	695	68.03
6	54	236	68.29	466	67.34	696	68.14
7	56.56	237	68.32	467	67.56	697	68.07
8	56.38	238	68.72	468	67.7	698	67.59
9	57.65	239	68.62	469	67.7	699	67.88
10	57.62	240	68.4	470	67.7	700	67.85
11	57.69	241	68.58	471	67.67	701	67.81
12	57.18	242	68.8	472	67.96	702	68.25
13	55.57	243	69.31	473	67.92	703	68.32
14	55.94	244	69.49	474	67.81	704	68.36
15	56.59	245	69.42	475	67.67	705	68.1
16	56.96	246	68.8	476	67.56	706	68.21
17	56.12	247	68.29	477	67.37	707	68.32
18	56.67	248	68.47	478	67.19	708	68.03
19	55.68	249	68.69	479	66.93	709	68.03
20	56.89	250	68.69	480	66.93	710	68.07
21	56.16	251	68.76	481	67.26	711	68.14
22	55.46	252	68.32	482	67.26	712	68.03
23	56.08	253	68.18	483	67.23	713	68.18
24	55.72	254	68.18	484	67.45	714	68.43
25	56.96	255	67.88	485	67.37	715	68.25
26	57.22	256	67.48	486	67.45	716	68.07
27	57.84	257	67.04	487	67.26	717	68.25
28	57.91	258	67.56	488	67.15	718	68.47
29	58.06	259	67.41	489	67.08	719	68.62
30	58.02	260	67.96	490	67.19	720	68.65
31	58.97	261	67.59	491	67.23	721	68.62
32	58.13	262	67.41	492	66.97	722	68.51
33	58.93	263	67.77	493	66.75	723	68.51
34	57.84	264	67.63	494	67.01	724	68.72
35	58.64	265	67.74	495	67.04	725	68.4
36	58.86	266	67.7	496	66.9	726	68.62
37	58.75	267	67.3	497	66.9	727	68.54
38	59.37	268	67.3	498	67.26	728	68.69
39	59.41	269	67.26	499	67.56	729	68.51
40	59.23	270	66.97	500	67.26	730	68.36
41	59.15	271	66.97	501	67.34	731	68.51
42	60.29	272	67.01	502	67.3	732	68.72
43	60.65	273	67.01	503	67.23	733	68.62
44	59.81	274	67.41	504	66.75	734	68.54
45	59.12	275	67.01	505	66.79	735	68.51
46	60.29	276	66.64	506	67.01	736	68.43
47	60.83	277	66.9	507	66.75	737	68.4

48	61.09	278	66.72	508	66.75	738	68.4
49	61.13	279	66.61	509	66.75	739	68.43
50	60.65	280	66.64	510	66.79	740	68.43
51	60.72	281	66.64	511	66.83	741	68.36
52	61.93	282	66.97	512	66.86	742	68.18
53	62.04	283	67.23	513	67.08	743	68.43
54	62.37	284	66.83	514	66.9	744	68.21
55	61.89	285	66.64	515	66.83	745	68.4
56	61.31	286	66.72	516	66.83	746	68.43
57	61.53	287	66.46	517	67.15	747	68.62
58	62.37	288	66.53	518	67.04	748	68.43
59	62.33	289	66.83	519	67.23	749	68.25
60	62.29	290	66.53	520	67.12	750	67.96
61	63.06	291	66.86	521	66.72	751	68.14
62	63.35	292	66.86	522	67.04	752	68.25
63	62.7	293	67.04	523	66.9	753	68.36
64	62.44	294	67.26	524	66.86	754	68.18
65	62	295	67.3	525	66.75	755	68.14
66	62.59	296	67.23	526	66.35	756	68.36
67	62.18	297	67.12	527	66.31	757	68.25
68	62.22	298	67.15	528	66.46	758	68.36
69	62.22	299	67.19	529	66.42	759	68.32
70	62.11	300	67.34	530	66.28	760	68.25
71	62.77	301	67.3	531	66.35	761	68.32
72	63.06	302	67.52	532	66.39	762	68.43
73	63.72	303	67.19	533	66.75	763	68.21
74	62.62	304	67.81	534	66.5	764	68.18
75	62.73	305	68.14	535	66.68	765	68.51
76	62.37	306	68.14	536	66.9	766	68.58
77	62.77	307	67.88	537	66.83	767	68.47
78	63.1	308	67.74	538	66.68	768	68.21
79	62.95	309	67.41	539	66.83	769	68.07
80	62.95	310	67.37	540	66.97	770	68.4
81	62.95	311	67.04	541	66.75	771	68.1
82	62.66	312	66.68	542	67.12	772	68.18
83	62.77	313	67.19	543	66.72	773	68.25
84	63.1	314	67.19	544	66.93	774	68.21
85	63.21	315	66.79	545	66.97	775	68.43
86	64.08	316	66.75	546	66.83	776	68.43
87	63.79	317	66.72	547	67.12	777	68.51
88	64.41	318	66.79	548	67.12	778	68.4
89	63.65	319	67.04	549	67.37	779	68.07
90	63.79	320	67.3	550	67.52	780	67.92
91	64.19	321	67.26	551	67.56	781	68.07
92	64.3	322	67.08	552	67.56	782	68.25
93	64.45	323	66.93	553	67.26	783	68.62
94	64.78	324	66.83	554	67.41	784	68.51
95	65.07	325	66.57	555	67.26	785	68.65
96	64.78	326	66.68	556	67.34	786	68.62
97	65.25	327	66.93	557	67.3	787	68.43
98	64.6	328	66.97	558	67.52	788	68.25
99	64.71	329	66.86	559	67.37	789	68.18
100	64.16	330	67.23	560	67.52	790	68.36
101	63.83	331	66.86	561	67.41	791	68.4

102	64.23	332	66.39	562	67.59	792	68.25
103	64.19	333	66.53	563	67.56	793	68.36
104	64.12	334	66.39	564	67.45	794	68.32
105	63.5	335	66.35	565	67.45	795	68.1
106	63.98	336	66.35	566	67.45	796	68.32
107	63.54	337	66.24	567	67.26	797	68.18
108	63.21	338	66.06	568	67.26	798	68.14
109	63.79	339	66.42	569	67.63	799	68.07
110	64.45	340	66.61	570	67.15	800	67.99
111	64.74	341	66.83	571	66.86	801	68.03
112	64.3	342	66.61	572	66.93	802	68.14
113	64.45	343	66.79	573	66.72	803	67.88
114	64.89	344	66.79	574	66.86	804	68.03
115	64.6	345	66.24	575	66.93	805	68.1
116	64.82	346	66.61	576	66.79	806	68.07
117	65.4	347	66.61	577	66.68	807	68.18
118	65.11	348	66.5	578	66.79	808	68.03
119	64.96	349	66.93	579	66.9	809	68.1
120	65	350	66.83	580	66.97	810	68.25
121	64.93	351	67.15	581	66.93	811	68.51
122	64.63	352	67.23	582	66.86	812	68.43
123	65.11	353	67.04	583	66.9	813	68.43
124	65.33	354	67.04	584	66.72	814	68.54
125	65.29	355	66.75	585	66.72	815	68.32
126	65.33	356	67.15	586	67.04	816	68.47
127	65.47	357	67.34	587	66.9	817	68.51
128	65.84	358	67.01	588	66.93	818	68.62
129	65.8	359	67.59	589	66.97	819	68.62
130	65.58	360	67.56	590	67.01	820	68.65
131	65.55	361	67.63	591	67.01	821	68.72
132	65.4	362	67.99	592	67.23	822	68.65
133	65.8	363	67.59	593	67.15	823	68.54
134	66.24	364	67.56	594	67.04	824	68.43
135	65.47	365	67.85	595	67.08	825	68.72
136	65	366	67.88	596	67.19	826	68.58
137	64.82	367	68.29	597	67.26	827	68.69
138	64.82	368	68.14	598	67.04	828	68.72
139	64.78	369	67.88	599	67.19	829	68.8
140	65.33	370	67.37	600	67.08	830	68.65
141	65.55	371	67.59	601	67.15	831	68.51
142	65.98	372	67.56	602	67.41	832	68.54
143	66.13	373	67.56	603	67.59	833	68.62
144	66.5	374	67.52	604	67.52	834	68.62
145	66.13	375	67.45	605	67.41	835	68.54
146	66.28	376	67.3	606	67.56	836	68.69
147	66.61	377	67.3	607	67.26	837	68.94
148	67.08	378	67.19	608	67.19	838	68.72
149	67.23	379	66.97	609	67.34	839	68.43
150	66.9	380	66.86	610	67.45	840	68.4
151	66.72	381	67.08	611	67.19	841	68.4
152	66.9	382	67.3	612	67.41	842	68.32
153	66.93	383	67.15	613	67.45	843	68.47
154	67.3	384	67.08	614	67.63	844	68.65
155	67.37	385	67.52	615	67.37	845	68.76

156	67.34	386	67.26	616	67.56	846	68.76
157	67.12	387	67.52	617	67.56	847	69.05
158	66.57	388	67.34	618	67.48	848	68.91
159	66.39	389	67.15	619	67.74	849	68.94
160	66.06	390	67.3	620	67.7	850	68.94
161	66.53	391	67.08	621	67.77	851	69.05
162	66.35	392	66.83	622	67.74	852	69.05
163	66.9	393	66.97	623	67.67	853	68.72
164	67.15	394	67.01	624	67.59	854	68.72
165	67.12	395	67.48	625	67.48	855	68.83
166	67.41	396	67.3	626	67.45	856	68.91
167	67.85	397	67.3	627	67.41	857	69.09
168	67.15	398	67.15	628	67.52	858	68.98
169	67.19	399	67.34	629	67.56	859	69.16
170	67.37	400	67.37	630	67.7	860	68.83
171	67.56	401	67.3	631	67.7	861	68.91
172	67.63	402	67.12	632	67.77	862	68.72
173	68.03	403	67.19	633	67.59	863	68.72
174	67.37	404	67.48	634	67.63	864	68.69
175	67.3	405	67.63	635	67.77	865	68.83
176	66.97	406	67.23	636	67.59	866	69.16
177	66.97	407	67.26	637	67.63	867	69.13
178	67.23	408	67.12	638	67.88	868	68.91
179	67.26	409	67.34	639	67.96	869	68.8
180	67.04	410	67.23	640	67.7	870	68.65
181	66.97	411	67.23	641	67.45	871	68.72
182	67.23	412	66.75	642	67.67	872	68.76
183	67.34	413	66.86	643	67.7	873	68.87
184	67.08	414	66.86	644	67.67	874	68.83
185	67.01	415	67.23	645	67.81	875	68.91
186	66.64	416	67.41	646	67.88	876	69.02
187	66.83	417	67.52	647	67.92	877	69.09
188	67.19	418	67.52	648	68.03	878	69.02
189	66.68	419	67.67	649	67.96	879	69.31
190	66.06	420	67.77	650	68.03	880	69.31
191	66.72	421	67.52	651	68.14	881	69.2
192	66.42	422	67.92	652	68.07	882	69.13
193	66.24	423	67.85	653	68.32	883	69.35
194	65.88	424	67.56	654	68.21	884	69.24
195	65.77	425	67.67	655	68.29	885	69.2
196	66.31	426	68.14	656	68.1	886	69.2
197	66.61	427	68.14	657	67.99	887	69.35
198	66.75	428	67.96	658	67.92	888	69.31
199	66.5	429	68.21	659	67.81	889	69.42
200	66.68	430	68.21	660	67.7	890	69.38
201	66.53	431	67.77	661	68.03	891	69.46
202	66.68	432	67.81	662	67.85	892	69.46
203	66.86	433	67.92	663	68.14	893	69.49
204	67.41	434	67.74	664	68.4	894	69.6
205	67.12	435	67.88	665	68.43	895	69.67
206	67.3	436	67.81	666	68.29	896	69.64
207	67.23	437	67.59	667	68.18	897	69.67
208	67.45	438	67.74	668	68.18	898	69.89
209	67.67	439	67.7	669	68.14	899	69.64

210	67.37	440	67.85	670	68.25	900	69.67
211	67.26	441	67.7	671	68.25	901	69.78
212	67.3	442	67.59	672	68.1	902	69.93
213	67.56	443	67.48	673	68.18	903	70.04
214	67.34	444	67.7	674	67.88	904	69.86
215	67.41	445	67.59	675	67.7	905	70.08
216	67.01	446	67.34	676	67.77	906	70.04
217	67.56	447	67.56	677	67.74	907	70
218	67.12	448	67.63	678	67.74	908	69.86
219	67.3	449	67.52	679	67.63	909	69.97
220	67.26	450	67.77	680	67.81	910	70.11
221	67.12	451	67.34	681	67.96	911	70.08
222	67.41	452	67.12	682	67.7	912	70.11
223	68.36	453	67.12	683	67.77	913	70.26
224	67.81	454	67.23	684	67.85	914	70.3
225	67.74	455	67.26	685	67.88	915	70.52
226	67.77	456	67.26	686	67.88	916	70.52
227	67.56	457	67.08	687	67.96	917	70.59
228	67.81	458	67.12	688	68.1	918	70.73
229	67.48	459	67.45	689	68.18		
230	67.12	460	67.34	690	68.14		
231	67.34	461	67.08	691	68.14		
