# Supplementary material for: Cooperativity among Short Amyloid Stretches in Long Amyloidogenic Sequences
Source: PLoS One. 2012 Jun 22;7(6):e39369. doi: 10.1371/journal.pone.0039369 (PMC3382238; doi:10.1371/journal.pone.0039369)
Supplement: Table S4 — The 446 features selected for feature analysis. (PDF) [file pone.0039369.s004.pdf]

Table S4. The 446 features selected for feature analysis.

List below are 446 features in optimal feature set that increase the accuracy.

Feature		
Order	Amino Acid	Attribute
1	AA26	Disorder
2	AA14	Propensity of amino acid to be conserved at protein
3	AA25	Pssm_H
4	AA14	Secondary Structure Strand
5	AA23	Pssm_H
6	AA13	Pssm_C
7	AA21	Pssm_H
8	AA13	Side Chain Count of Atom_C Deviation from Mean
9	AA15	Gain/loss of amino acids during evolution
10	AA12	Pssm_V
11	AA2	Propensity of amino acid to be conserved at protein
12	AA16	Pssm_V
13	AA24	Solvent Accessibility Buried
14	AA7	Pssm_Y
15	AA6	Molecular Volume
16	AA11	Pssm_C
17	AA6	Pssm_C
18	AA24	Gain/loss of amino acids during evolution
19	AA14	Gain/loss of amino acids during evolution
20	AA7	Pssm_C
21	AA2	Secondary Structure Helix
22	AA5	Molecular Volume
23	AA17	Propensity of amino acid to be conserved at protein
24	AA18	Pssm_H
25	AA14	Pssm_C
26	AA4	Pssm_T
27	AA14	Molecular Volume
28	AA17	Pssm_Y
29	AA15	Disorder
30	AA20	Molecular Volume
31	AA7	Pssm_T
32	AA12	Molecular Volume
33	AA14	Pssm_M
34	AA2	Pssm_T
35	AA24	Pssm_R
36	AA13	Pssm_H
37	AA17	Gain/loss of amino acids during evolution
38	AA26	Gain/loss of amino acids during evolution
39	AA8	Pssm_C
40	AA15	Polarity
41	AA2	Pssm_A
42	AA12	Pssm_M
43	AA7	Propensity of amino acid to be conserved at protein

44	AA15	Pssm_Y
45	AA2	Pssm_L
46	AA11	Pssm_T
47	AA14	Pssm_S
48	AA10	Secondary Structure Strand
49	AA1	Pssm_V
50	AA18	Secondary Structure Strand
51	AA4	Pssm_S
52	AA4	Pssm_C
53	AA21	Disorder
54	AA3	Pssm_N
55	AA1	Pssm_W
56	AA7	Molecular Volume
57	AA26	Pssm_Y
58	AA15	Electrostatic Charge
59	AA6	Pssm_R
60	AA26	Solvent Accessibility Buried
61	AA20	Secondary Structure Strand
62	AA10	Pssm_H
63	AA19	Pssm_D
64	AA9	Pssm_H
65	AA4	Pssm_H
66	AA10	Molecular Volume
67	AA19	Pssm_M
68	AA15	Pssm_I
69	AA4	Pssm_V
70	AA24	Pssm_C
71	AA1	Secondary Structure Strand
72	AA15	Pssm_H
73	AA12	Pssm_T
74	AA24	Polarity
75	AA3	Pssm_R
76	AA19	Pssm_R
77	AA4	Polarity
78	AA2	Electrostatic Charge
79	AA13	Secondary Structure
80	AA21	Pssm_A
81	AA26	Pssm_E
82	AA22	Pssm_H
83	AA10	Pssm_V
84	AA19	Pssm_C
85	AA6	Pssm_N
86	AA16	Pssm_A
87	AA9	Polarity
88	AA16	Secondary Structure Strand
89	AA13	Gain/loss of amino acids during evolution
90	AA16	Molecular Volume
91	AA17	Secondary Structure Other

92	AA10	Pssm_I
93	AA16	Pssm_E
94	AA25	Gain/loss of amino acids during evolution
95	AA23	Pssm_C
96	AA27	Pssm_L
97	AA13	Pssm_T
98	AA23	Pssm_M
99	AA20	Pssm_M
100	AA15	Pssm_S
101	AA11	Pssm_S
102	AA26	Pssm_Q
103	AA15	Secondary Structure
104	AA5	Pssm_K
105	AA1	Pssm_D
106	AA16	Pssm_T
107	AA25	Solvent Accessibility Buried
108	AA15	Side Chain Count of Atom_C Deviation from Mean
109	AA10	Propensity of amino acid to be conserved at protein
110	AA5	Pssm_V
111	AA4	Side Chain Count of Atom_C Deviation from Mean
112	AA2	Pssm_Q
113	AA4	Pssm_R
114	AA16	Secondary Structure Other
115	AA7	Pssm_K
116	AA7	Pssm_F
117	AA26	Secondary Structure Helix
118	AA1	Pssm_P
119	AA9	Secondary Structure Helix
120	AA26	Secondary Structure Strand
121	AA23	Pssm_V
122	AA27	Disorder
123	AA2	Pssm_K
124	AA26	Polarity
125	AA20	Gain/loss of amino acids during evolution
126	AA7	Pssm_V
127	AA3	Pssm_A
128	AA24	Pssm_A
129	AA15	Secondary Structure Other
130	AA5	Secondary Structure Strand
131	AA7	Pssm_M
132	AA7	Codon Diversity
133	AA27	Pssm_Q
134	AA27	Secondary Structure Other
135	AA20	Pssm_I
136	AA10	Secondary Structure Other
137	AA3	Pssm_C
138	AA21	Pssm_T
139	AA3	Pssm_F

140	AA10	Solvent Accessibility Exposed
141	AA25	Pssm_S
142	AA21	Pssm_S
143	AA1	Disorder
144	AA27	Propensity of amino acid to be conserved at protein
145	AA9	Pssm_A
146	AA6	Pssm_G
147	AA15	Pssm_F
148	AA27	Gain/loss of amino acids during evolution
149	AA13	Molecular Volume
150	AA8	Pssm_T
151	AA27	Pssm_H
152	AA11	Molecular Volume
153	AA22	Pssm_M
154	AA5	Polarity
155	AA4	Electrostatic Charge
156	AA23	Pssm_S
157	AA9	Disorder
158	AA1	Pssm_H
159	AA8	Pssm_V
160	AA26	Pssm_R
161	AA20	Electrostatic Charge
162	AA1	Pssm_R
163	AA11	Pssm_I
164	AA10	Pssm_G
165	AA17	Pssm_P
166	AA16	Pssm_C
167	AA2	Molecular Volume
168	AA5	Side Chain Count of Atom_C Deviation from Mean
169	AA17	Pssm_C
170	AA15	Pssm_T
171	AA13	Pssm_R
172	AA7	Pssm_H
173	AA17	Secondary Structure Strand
174	AA27	Pssm_R
175	AA25	Pssm_N
176	AA6	Pssm_M
177	AA7	Pssm_L
178	AA17	Codon Diversity
179	AA10	Secondary Structure
180	AA12	Secondary Structure
181	AA21	Pssm_W
182	AA6	Pssm_F
183	AA27	Pssm_P
184	AA2	Solvent Accessibility Exposed
185	AA13	Pssm_S
186	AA9	Pssm_I
187	AA12	Secondary Structure Other

188	AA3	Pssm_K
189	AA18	Pssm_P
190	AA19	Secondary Structure Helix
191	AA9	Gain/loss of amino acids during evolution
192	AA24	Pssm_Y
193	AA27	Molecular Volume
194	AA3	Side Chain Count of Atom_C Deviation from Mean
195	AA23	Pssm_T
196	AA5	Pssm_N
197	AA26	Propensity of amino acid to be conserved at protein
198	AA9	Codon Diversity
199	AA21	Pssm_D
200	AA14	Secondary Structure Other
201	AA14	Pssm_N
202	AA22	Pssm_R
203	AA6	Secondary Structure
204	AA1	Pssm_Q
205	AA23	Pssm_K
206	AA21	Pssm_N
207	AA27	Pssm_S
208	AA3	Pssm_D
209	AA26	Pssm_I
210	AA16	Pssm_D
211	AA7	Pssm_P
212	AA3	Pssm_Q
213	AA16	Polarity
214	AA1	Side Chain Count of Atom_C Deviation from Mean
215	AA8	Pssm_N
216	AA5	Pssm_I
217	AA17	Pssm_K
218	AA1	Pssm_K
219	AA13	Pssm_D
220	AA17	Pssm_T
221	AA2	Disorder
222	AA26	Side Chain Count of Atom_C Deviation from Mean
223	AA23	Polarity
224	AA5	Secondary Structure
225	AA9	Secondary Structure Other
226	AA17	Pssm_V
227	AA18	Pssm_M
228	AA24	Electrostatic Charge
229	AA8	Secondary Structure
230	AA5	Pssm_F
231	AA9	Pssm_K
232	AA26	Pssm_S
233	AA18	Pssm_S
234	AA11	Gain/loss of amino acids during evolution
235	AA25	Propensity of amino acid to be conserved at protein

236	AA20	Pssm_R
237	AA2	Codon Diversity
238	AA11	Secondary Structure
239	AA22	Electrostatic Charge
240	AA15	Pssm_K
241	AA1	Pssm_F
242	AA15	Solvent Accessibility Buried
243	AA7	Secondary Structure Other
244	AA6	Secondary Structure Other
245	AA12	Secondary Structure Helix
246	AA17	Secondary Structure Helix
247	AA8	Disorder
248	AA27	Solvent Accessibility Buried
249	AA17	Pssm_H
250	AA11	Secondary Structure Strand
251	AA23	Pssm_Q
252	AA1	Pssm_A
253	AA25	Disorder
254	AA1	Pssm_N
255	AA8	Pssm_D
256	AA12	Pssm_F
257	AA20	Pssm_N
258	AA27	Secondary Structure Helix
259	AA18	Pssm_L
260	AA7	Pssm_I
261	AA4	Pssm_I
262	AA22	Pssm_K
263	AA6	Pssm_K
264	AA18	Pssm_K
265	AA5	Disorder
266	AA25	Pssm_Y
267	AA7	Pssm_R
268	AA16	Pssm_N
269	AA13	Pssm_M
270	AA24	Pssm_F
271	AA16	Codon Diversity
272	AA24	Pssm_D
273	AA26	Solvent Accessibility Exposed
274	AA8	Solvent Accessibility Buried
275	AA13	Solvent Accessibility Exposed
276	AA13	Pssm_W
277	AA7	Pssm_W
278	AA15	Pssm_G
279	AA13	Secondary Structure Helix
280	AA13	Pssm_V
281	AA11	Pssm_P
282	AA3	Polarity
283	AA26	Pssm_L

284	AA17	Pssm_A
285	AA20	Pssm_T
286	AA23	Solvent Accessibility Buried
287	AA3	Electrostatic Charge
288	AA3	Pssm_I
289	AA3	Pssm_P
290	AA13	Disorder
291	AA22	Pssm_V
292	AA12	Pssm_R
293	AA17	Molecular Volume
294	AA15	Pssm_R
295	AA22	Pssm_T
296	AA12	Polarity
297	AA27	Codon Diversity
298	AA10	Solvent Accessibility Buried
299	AA6	Codon Diversity
300	AA4	Pssm_W
301	AA24	Secondary Structure Strand
302	AA16	Pssm_I
303	AA4	Pssm_L
304	AA6	Secondary Structure Helix
305	AA15	Pssm_Q
306	AA25	Pssm_D
307	AA23	Pssm_I
308	AA3	Solvent Accessibility Buried
309	AA11	Pssm_D
310	AA10	Pssm_S
311	AA13	Pssm_Q
312	AA23	Pssm_D
313	AA8	Pssm_W
314	AA11	Pssm_G
315	AA27	Pssm_K
316	AA20	Secondary Structure Other
317	AA19	Pssm_Q
318	AA17	Pssm_G
319	AA17	Pssm_Q
320	AA19	Pssm_K
321	AA22	Pssm_E
322	AA23	Pssm_E
323	AA24	Secondary Structure Helix
324	AA19	Pssm_P
325	AA3	Disorder
326	AA20	Pssm_P
327	AA21	Pssm_C
328	AA3	Pssm_T
329	AA4	Secondary Structure Strand
330	AA19	Pssm_H
331	AA1	Pssm_T

332	AA12	Pssm_Y
333	AA5	Secondary Structure Helix
334	AA8	Pssm_M
335	AA3	Secondary Structure Helix
336	AA7	Solvent Accessibility Exposed
337	AA10	Pssm_K
338	AA22	Pssm_A
339	AA9	Pssm_R
340	AA16	Pssm_S
341	AA7	Solvent Accessibility Buried
342	AA17	Pssm_S
343	AA24	Disorder
344	AA27	Pssm_W
345	AA16	Pssm_Q
346	AA6	Solvent Accessibility Buried
347	AA14	Pssm_E
348	AA2	Pssm_P
349	AA9	Pssm_Q
350	AA21	Pssm_K
351	AA4	Pssm_P
352	AA19	Pssm_E
353	AA8	Pssm_P
354	AA5	Propensity of amino acid to be conserved at protein
355	AA25	Side Chain Count of Atom_C Deviation from Mean
356	AA19	Pssm_T
357	AA18	Electrostatic Charge
358	AA6	Pssm_D
359	AA18	Pssm_R
360	AA7	Pssm_Q
361	AA18	Solvent Accessibility Exposed
362	AA14	Pssm_D
363	AA12	Pssm_I
364	AA1	Pssm_I
365	AA5	Pssm_W
366	AA10	Pssm_P
367	AA22	Gain/loss of amino acids during evolution
368	AA23	Pssm_A
369	AA18	Pssm_N
370	AA8	Secondary Structure Other
371	AA4	Pssm_F
372	AA26	Pssm_N
373	AA13	Secondary Structure Other
374	AA1	Solvent Accessibility Buried
375	AA22	Solvent Accessibility Exposed
376	AA2	Pssm_I
377	AA5	Pssm_G
378	AA18	Pssm_I
379	AA22	Pssm_P

380	AA23	Pssm_P
381	AA10	Disorder
382	AA19	Secondary Structure
383	AA9	Molecular Volume
384	AA24	Molecular Volume
385	AA17	Polarity
386	AA24	Pssm_T
387	AA18	Pssm_A
388	AA4	Secondary Structure
389	AA14	Pssm_F
390	AA25	Pssm_W
391	AA20	Polarity
392	AA2	Side Chain Count of Atom_C Deviation from Mean
393	AA22	Disorder
394	AA8	Side Chain Count of Atom_C Deviation from Mean
395	AA15	Codon Diversity
396	AA26	Codon Diversity
397	AA25	Pssm_L
398	AA21	Secondary Structure
399	AA19	Pssm_F
400	AA17	Solvent Accessibility Exposed
401	AA24	Secondary Structure Other
402	AA26	Pssm_W
403	AA12	Disorder
404	AA8	Propensity of amino acid to be conserved at protein
405	AA21	Pssm_V
406	AA4	Pssm_N
407	AA21	Molecular Volume
408	AA1	Codon Diversity
409	AA1	Pssm_S
410	AA19	Pssm_W
411	AA2	Pssm_D
412	AA11	Pssm_K
413	AA6	Pssm_S
414	AA25	Secondary Structure Other
415	AA10	Secondary Structure Helix
416	AA18	Disorder
417	AA15	Pssm_P
418	AA25	Pssm_C
419	AA13	Pssm_Y
420	AA13	Secondary Structure Strand
421	AA14	Pssm_Y
422	AA14	Electrostatic Charge
423	AA7	Pssm_A
424	AA9	Secondary Structure
425	AA27	Pssm_T
426	AA2	Secondary Structure
427	AA19	Pssm_A

428	AA5	Solvent Accessibility Exposed
429	AA19	Pssm_N
430	AA23	Secondary Structure Other
431	AA9	Pssm_F
432	AA27	Solvent Accessibility Exposed
433	AA10	Pssm_L
434	AA3	Pssm_E
435	AA12	Pssm_D
436	AA17	Pssm_F
437	AA15	Pssm_L
438	AA21	Solvent Accessibility Exposed
439	AA22	Pssm_D
440	AA24	Pssm_I
441	AA19	Pssm_G
442	AA8	Pssm_E
443	AA25	Pssm_E
444	AA24	Pssm_E
445	AA14	Secondary Structure Helix
446	AA6	Disorder
