# Supplementary material for: Cooperativity among Short Amyloid Stretches in Long Amyloidogenic Sequences
Source: PLoS One. 2012 Jun 22;7(6):e39369. doi: 10.1371/journal.pone.0039369 (PMC3382238; doi:10.1371/journal.pone.0039369)
Supplement: Text S1 — The detailed description of the methods used in this research. (PDF) [file pone.0039369.s005.pdf]

Cooperativities Among Short Amyloid Stretches Within Long Amyloidogenic Sequence Segment

Lele Hu[1,3,#], Weiren Cui[2,#], Zhisong He[2], Xiaohe Shi[4], Kaiyan Feng[5], Ruth Nussinov[6], Buyong Ma[6,*] Yu-Dong Cai[1,*]

[1]Institute of Systems Biology, Shanghai University, Shanghai 200444, P. R. China
[2]CAS-MPG Partner Institute of Computational Biology, Shanghai Institutes for Biological Sciences, Chinese Academy of Sciences, Shanghai 200031, P. R. China
[3]Department of Chemistry, College of Sciences, Shanghai University, Shanghai, P.R. China
[4]Institute of Health Sciences, Shanghai Institutes for Biological Sciences, Chinese Academy of Sciences and Shanghai Jiao Tong University School of Medicine, Shanghai 200025, P. R. China
[5]Shanghai Center for Bioinformation Technology, Shanghai 200235, China
[6] Basic Science Program, SAIC – Frederick, Center for Cancer Research Nanobiology Program, NCI-Fredeick, NIH, Frederick, MD 21702, USA

[#] These authors contribute equally.

[*] To whom correspondence should be addressed.

Buyong Ma, Email: mabuyong@mail.nih.gov, Tel: 001-301-8466540

Yu-Dong Cai, Email: cai_yud@yahoo.com.cn, Tel: 0086-21-66136132

Material and Methods

1. Dataset

Both of the positive and negative training data were constructed from the previous computational approaches (1-3). With the fibril-forming segments these works provided, the original sequences of the whole proteins were obtained by searching the peptide sequences in the SwissProt database, only the perfect matches with the same sequence information, protein names and the organisms were selected. Finally, 46 protein sequences with 17102 amino acids were obtained, in which there are 1370 fibril-forming sites according to the laboratory work. A scanning window of 27 amino acids wide was used to generate the training peptides. These peptides with 27 amino acids had its category due to the location of the 14th amino acid. If the 14th amino acid located in the fibril-forming region, the peptide was sorted as the positive data, while the peptide with the non-fibril-forming 14th amino acid was classified as the negative data. As the negative data is 11 times larger than the positive one, and the unbalanced dataset between the negative sample and positive sample may cause bias during the training process, a random selection were carried out to construct a balanced dataset which contains 1370 positive data and 1370 negative data.

2 Feature Construction

2.1 The features of AAIndex transformed scores

AAIndex (http://www.genome.ad.jp/aaindex/) is a database of numerical indices representing various physicochemical and biochemical properties of amino acids and pairs of amino acids. It is composed of three sections: AAIndex1, AAIndex2 and AAIndex3. AAindex1 is the database for 544 different physicochemical and biological properties of amino acids. While these properties in AAindex1 were

summarized into 5 multidimensional patterns of attribute covariation by Atchley et al in 2005 (4). The 5 transformed scores used to encode the amino acids in our study shows the properties of polarity, secondary structure, molecular volume, codon diversity, and electrostatic charge individually.

2.2 The features of disorder score

Disordered regions are parts of the proteins which don't have fixed three-dimensional structures under physiological conditions. They involve high-specificity low affinity interactions and multiple binding of proteins, and is very important in regulation, signaling and control (5).

VSL2 (6) was used to calculate the disorder score which quantifies the disorder status of each amino acid in the protein sequence. The VSL2 predictors can accurately predict and identify the short disordered regions. The disorder scores of all the 9 amino acids in the training peptides were included in the features that encoded the peptide.

2.3 The features of PSSM conservation score

Conservation is one of the most important concepts in biology. It's known that there are many conservative sequences among different species, while these sequences haven't changed a lot during the evolution, and often locate in the important functional domains. This means that these amino acid sites are of great importance, and the mutation at these sites is able to result in the change of the structure or function of the protein.

Position Specific Iterative BLAST (PSI BLAST) can measure the residue conservation in a given location. It uses a 20-dimensional vector to represent probabilities of conservation against mutations to 20 different amino acids. Position Specific Scoring Matrix (PSSM)(7) is a matrix of such vectors which represent all

residues in a given sequence. If a residue is conserved in PSI BLAST, it is likely to be important for biological function.

In this study, we used the PSSM conservation score to quantify the conservation status of each amino acid in the protein sequence. Target sequences are scanned against the reference data sets UniRef100 (Release: 15.9, 13-Oct-2009) to generate the position specific scoring matrices (PSSMs) using Position Specific Iterative BLAST (PSI BLAST) program (Release 2.2.12)(8).

2.4 The features of secondary structure and solvent accessibility

The basic functions of proteins are mainly controlled by their structure. Here, the structure features including secondary and solvent accessibility were also included to encode the peptides. These features were predicted by the secondary structure and solvent accessibility predictors SSpro 4 (9). The secondary structural property of each amino acid was predicted as ‘helix’, ‘strand’, or ‘other’. And the solvent accessibility of each amino acid was predicted as ‘buried’ or ‘exposed’.

2.5 The Other features

Goldsmidt et al suggested that protein folds have evolved to remove segments of high propensity and proper conformation for fibrillation from protein surfaces(10). Consistently, we included features of amino acid evolution (11), the conservation of an amino acid on protein exposed surface (12). Besides, we also included the side chain count of carbon atom deviation from mean for each amino acid residue in the peptide for the atoms composition of side chain influence the structures and functions of the proteins. The score for each residue can be calculated by subtracting the mean of the carbon atom number of residue side chains within the 27-residue peptide by the side chain carbon number of each residue.

2. Nearest Neighbor Algorithm

In this study, Nearest Neighbor (NN) algorithm (13-16) was used to construct classifiers to classify each sample to a fibril-forming one or a non-fibril-forming one. NN is a machine learning approach which has been widely applied in biological researches (13-16). It makes its decision based on similarities between the test sample's feature vector and the feature vectors of all samples in training dataset. The same class of the sample in the training set which has the highest similarity, i.e. the nearest neighbor, would be designated to the test sample. In this study, the similarity between two vectors p_i and p_j is defined as:

$$D(p_i, p_j) = \frac{p_i \cdot p_j}{\| p_i \| \cdot \| p_j \|}$$

where $p_i \cdot p_j$ is the inner product of p_i and p_j, and $\|p\|$ represents the module of vector p.

3. Random forest algorithm

Besides the NN algorithm, random forest (RF) algorithm (17) was also used to construct classifier for it has been successfully applied in the diverse biological prediction problems (18-20). RF classifier consists of many decision trees and makes decisions by choosing the class with the most votes of the decision trees in the forest. Each tree can be grown like this: (1) Suppose the number of training samples is N, and the number of variables in the classifier is M. (2) Then a training set is generated for the tree by choosing n times with replacement from all the N training samples (Bootstrap aggregating), and the rest samples are used for test. (3) For each node of the tree, m (much less than M) variables are chose randomly from the M variables to make decisions. Based on the selected m variables, the most optimized split is employed to split the node. (4) Each tree should be fully grown with no pruning. By combining multiple trees produced in randomly selected subspaces, the prediction accuracy is shown to be significantly improved. The detailed implement of the algorithm can be found in the researches (17).

a. Jackknife Cross-Validation Method

Jackknife Cross-Validation Method(13, 21) is thought as one of the most effective and objective ways to evaluate statistical predictions. In Jackknife Cross-Validation Method, each sample in the data set is knocked out and tested by the predictor trained by the other samples in the data set. During this process, each sample not only involves in the test set, but also the training set. To evaluate the performance of a predictor, the accurate rate for positive samples, negative samples and the overall accurate rate will be used:

$$\begin{cases} \text{accuracy of positive dataset} = \dfrac{\text{correctly predicted true samples}}{\text{true samples}} \\ \text{accuracy of negative dataset} = \dfrac{\text{correctly predicted false samples}}{\text{false samples}} \\ \text{overall accuracy} = \dfrac{\text{correctly predicted true sampless} + \text{correctly predicted false samples}}{\text{true samples} + \text{false samples}} \end{cases}$$

b. Feature Selection

i. mRMR method for feature evaluation

Maximum Relevance, Minimum Redundancy method(22) is originally developed by Peng *et al*. It ranks each feature according to both its relevance to the target (highly related to the prediction accuracy) and the redundancy between the features. A “good” feature is characterized by maximum relevance with the target variable and minimum redundancy within the features. Mutual information (MI), which estimates how much one vector is related to another, is used to quantify the relevance and redundancy of each feature. MI is defined as follows:

$$I(x,y) = \iint p(x,y)\log\frac{p(x,y)}{p(x)p(y)}dxdy \qquad (1)$$

where x and y are two vectors; $p(x,y)$ is the joint probabilistic density;

$p(x)$ and $p(y)$ are the marginal probabilistic densities.

The mRMR function, that maximizes relevance and minimize redundancy, is defined as:

$$\max_{f_j \in \Omega_t} \left[I(f_j, c) - \frac{1}{m} \sum_{f_i \in \Omega_s} I(f_{j,} f_i) \right] (j = 1, 2, ..., n) \qquad (2)$$

Where Ω_s is the already-selected vector set with m vectors, Ω_t is the to-be-selected vector set with n vectors. $D = I(f, c)$ is the relevance of a feature f in Ω_t with a classification variable c, while $R = \frac{1}{m} \sum_{f_i \in \Omega_s} I(f, f_i)$ is the redundancy of a feature f in Ω_t with all the features in Ω_s

For a feature pool containing N (N = m + n) features, feature evaluation will continue N rounds. After the pre-evaluation procedure, mRMR method will provide us a feature set S:

$$S = \left[f_0^{'}, f_1^{'}, ..., f_h^{'}, ..., f_{N-1}^{'} \right] \qquad (3)$$

In the feature set S, the feature index h denotes which round the feature is selected at. Evaluations for feature are also reflected by these indices. For example, f_a is believed better than f_b, if a < b, because the better the feature satisfies Eq (2), the earlier it will be added to S.

ii. Incremental Feature Selection (IFS)

With the mRMR result, we know the order of the features from the best feature to the worst feature. In order to get the optimal feature set which contains the optimal number of the features, Incremental Feature Selection (IFS) was used.

In mRMR step, we can construct the N feature sets from ordered feature set S, where the *i*-th feature set is:

$$S_i = \{ f_0, f_1, ..., f_i \} (0 \le i \le N-1) \qquad (4)$$

For every *i* between 0 and *N*-1, we use NN or RF to construct the predictor with the feature set S_i. Jackknife Cross-Validation Method is then used to obtain the accurate rates. As a result, we can get an IFS curve with index *i* as its x-axis and the overall accurate rate as its y-axis.

References

1. Fernandez-Escamilla AM, Rousseau F, Schymkowitz J, & Serrano L (2004) Prediction of sequence-dependent and mutational effects on the aggregation of peptides and proteins. *Nat Biotechnol* 22(10):1302-1306.
2. Tian J, Wu N, Guo J, & Fan Y (2009) Prediction of amyloid fibril-forming segments based on a support vector machine. *BMC Bioinformatics* 10 Suppl 1:S45.
3. Maurer-Stroh S, *et al.* (Exploring the sequence determinants of amyloid structure using position-specific scoring matrices. *Nat Methods* 7(3):237-242.
4. Atchley WR, Zhao J, Fernandes AD, & Druke T (2005) Solving the protein sequence metric problem. *Proc Natl Acad Sci U S A* 102(18):6395-6400.
5. Sickmeier M, *et al.* (2007) DisProt: the Database of Disordered Proteins. (Translated from eng) *Nucleic Acids Res* 35(Database issue):D786-793 (in eng).
6. Peng K, Radivojac P, Vucetic S, Dunker AK, & Obradovic Z (2006) Length-dependent prediction of protein intrinsic disorder. (Translated from eng) *BMC Bioinformatics* 7:208 (in eng).
7. Ahmad S & Sarai A (2005) PSSM-based prediction of DNA binding sites in proteins. (Translated from eng) *BMC Bioinformatics* 6:33 (in eng).
8. Altschul SF, *et al.* (1997) Gapped BLAST and PSI-BLAST: a new generation of protein database search programs. *Nucleic Acids Res* 25(17):3389-3402.
9. Cheng J, Randall AZ, Sweredoski MJ, & Baldi P (2005) SCRATCH: a protein structure and structural feature prediction server. (Translated from eng) *Nucleic acids research* 33(Web Server issue):W72-76 (in eng).
10. Goldschmidt L, Teng PK, Riek R, & Eisenberg D (2010) Identifying the amylome, proteins capable of forming amyloid-like fibrils. *Proc Natl Acad Sci U S A* 107(8):3487-3492.
11. Jordan IK, *et al.* (2005) A universal trend of amino acid gain and loss in protein evolution. *Nature* 433(7026):633-638.
12. **Ma B**, Elkayam T, Wolfson H, & Nussinov R (2003) Protein-protein interactions: structurally conserved residues distinguish between binding sites and exposed protein surfaces. *Proc Natl Acad Sci U S A* 100(10):5772-5777.
13. Cai Y, *et al.* (2009) A novel computational approach to predict transcription factor DNA binding preference. (Translated from eng) *J Proteome Res* 8(2):999-1003 (in eng).
14. Cai YD, *et al.* (2008) Prediction of compounds' biological function (metabolic pathways) based on functional group composition. (Translated from eng) *Mol Divers* 12(2):131-137 (in eng).
15. Tartaglia GG, *et al.* (2008) Prediction of aggregation-prone regions in structured proteins. *J Mol Biol* 380(2):425-436.
16. Lu L, *et al.* (2009) Protein sumoylation sites prediction based on two-stage feature selection. (Translated from Eng) *Mol Divers* (in Eng).
17. Breiman L (2001) Random Forests. *Mach. Learn.* 45(1):5-32.
18. Jia SC & Hu XZ (2011) Using random forest algorithm to predict beta-hairpin motifs. (Translated from eng) *Protein Pept Lett* 18(6):609-617 (in eng).
19. Kandaswamy KK, *et al.* (2011) AFP-Pred: A random forest approach for predicting antifreeze proteins from sequence-derived properties. (Translated from eng) *J Theor Biol* 270(1):56-62 (in eng).
20. Lin WZ, Fang JA, Xiao X, & Chou KC (2011) iDNA-Prot: identification of DNA binding proteins using random forest with grey model. (Translated from eng) *Plos One* 6(9):e24756 (in eng).

21. Hamodrakas SJ, Liappa C, & Iconomidou VA (2007) Consensus prediction of amyloidogenic determinants in amyloid fibril-forming proteins. *Int J Biol Macromol* 41(3):295-300.
22. Peng H, Long F, & Ding C (2005) Feature selection based on mutual information: criteria of max-dependency, max-relevance, and min-redundancy. (Translated from eng) *IEEE Trans Pattern Anal Mach Intell* 27(8):1226-1238 (in eng).
